# CXCL13 is expressed in a subpopulation of neuroendocrine cells in the murine trachea and lung

**DOI:** 10.1007/s00441-021-03552-2

**Published:** 2021-11-11

**Authors:** Wafaa Mahmoud, Alexander Perniss, Krupali Poharkar, Aichurek Soultanova, Uwe Pfeil, Andreas Hoek, Sudhanshu Bhushan, Torsten Hain, Ulrich Gärtner, Wolfgang Kummer

**Affiliations:** 1 Institute for Anatomy and Cell Biology, German Center for Lung Research, Excellence Cluster Cardio-Pulmonary Institute (CPI), Justus Liebig University Giessen, 35392 Giessen, Germany; 2 Department of Anatomy, Faculty of Medicine, Jordan University of Science and Technology, P.O. Box 3030, Irbid, 22110 Jordan; 3 Institute for Bioinformatics and Systems Biology, Justus Liebig University Giessen, 35392 Giessen, Germany; 4 Institute for Anatomy and Cell Biology, Unit of Reproductive Biology, Justus Liebig University Giessen, 35392 Giessen, Germany; 5 Institute of Medical Microbiology, German Center for Infection Research, Partner Site Giessen-Marburg-Langen, Justus Liebig University Giessen, 35392 Giessen, Germany

**Keywords:** Airway epithelium, Neuroendocrine cells, Neuroepithelial bodies, CXCL13, Innate immunity, BALT

## Abstract

The conducting airways are lined by distinct cell types, comprising basal, secretory, ciliated, and rare cells, including ionocytes, solitary cholinergic chemosensory cells, and solitary and clustered (neuroepithelial bodies) neuroendocrine cells. Airway neuroendocrine cells are in clinical focus since they can give rise to small cell lung cancer. They have been implicated in diverse functions including mechanosensation, chemosensation, and regeneration, and were recently identified as regulators of type 2 immune responses via the release of the neuropeptide calcitonin gene-related peptide (CGRP). We here assessed the expression of the chemokine CXCL13 (B cell attracting chemokine) by these cells by RT-PCR, in silico analysis of publicly available sequencing data sets, immunohistochemistry, and immuno-electron microscopy. We identify a phenotype of neuroendocrine cells in the naïve mouse, producing the chemokine CXCL13 predominantly in solitary neuroendocrine cells of the tracheal epithelium (approx. 70% CXCL13^+^) and, to a lesser extent, in the solitary neuroendocrine cells and neuroepithelial bodies of the intrapulmonary bronchial epithelium (< 10% CXCL13^+^). In silico analysis of published sequencing data of murine tracheal epithelial cells was consistent with the results obtained by immunohistochemistry as it revealed that neuroendocrine cells are the major source of *Cxcl13*-mRNA, which was expressed by 68–79% of neuroendocrine cells. An unbiased scRNA-seq data analysis of overall gene expression did not yield subclusters of neuroendocrine cells. Our observation demonstrates phenotypic heterogeneity of airway neuroendocrine cells and points towards a putative immunoregulatory role of these cells in bronchial-associated lymphoid tissue formation and B cell homeostasis.

**Supplementary Information:**

The online version contains supplementary material available at 10.1007/s00441-021-03552-2.

## Introduction

Neuroendocrine cells, cholinergic chemosensory cells, and ionocytes are rare epithelial cell types of the airways (Plasschaert et al. 2018; Montoro et al. 2018). Cholinergic chemosensory cells are sentinels of microbial products initiating protective neural reflexes and local innate immune responses (Krasteva et al. 2011, 2012; Perniss et al. 2020); ionocytes exhibit particularly high expression of the ion channel CFTR (cystic fibrosis transmembrane regulator) and might be involved in the pathogenesis of cystic fibrosis (Montoro et al. 2018; Plasschaert et al. 2018). Neuroendocrine cells raised clinical interest since they can give rise to small cell lung cancer (Hattori et al. 1972; Park et al. 2011). They occur as solitary neuroendocrine cells, dispersed in the epithelium of the conducting lower airways from the trachea down to the bronchioles, and in clusters termed neuroepithelial bodies, located mainly at branching points of intrapulmonary airways. Their distinctive structural feature is secretory dense core granules, which accumulate in the basal region of the cell. Secretory products of airway neuroendocrine cells are, with some species-specific variations, different amines, such as serotonin and γ-aminobutyric acid (GABA), and neuropeptides, such as calcitonin gene-related peptide (CGRP) (Cutz et al. 2013). Neuroepithelial bodies have been implicated with diverse functions including roles as stem cells promoting epithelial regeneration after injury (Ouadah et al. 2019; Reynolds et al. 2000; Song et al. 2012), mechano- (Lembrechts et al. 2012), and hypoxia sensors (Fu et al. 2000; Kazemian et al. 2001). Recent work identified them as crucial regulators of type 2 immune response in the airways. Mutant mice lacking neuroendocrine cells due to a genetic deficiency of the transcription factor *Ascl1* exhibit severely blunted mucosal type 2 response in models of allergic asthma (Sui et al. 2018). In mouse models of allergic airway inflammation, GABA released from neuroepithelial bodies induces goblet cell hyperplasia, and CGRP stimulates group 2 innate lymphoid cells to release IL-5, which in the end recruits eosinophils (Sui et al. 2018).

Immune cell recruitment in allergic airway inflammation is not restricted to eosinophils but also includes B cells and the formation of bronchus-associated lymphoid tissue (BALT) (Chvatchko et al. 1996). The chemokine (C-X-C motif) ligand 13 (CXCL13), also known as B cell-attracting chemokine 1 (BCA-1), is indispensable for this process. CXCL13 attracts B lymphocytes through binding to the G protein-coupled receptor CXCR5/BLR1, which is highly expressed not only by B cells, but also by a subpopulation of CD4^+^ and CD8^+^ T helper cells (Legler and Thelen 2016; Förster et al. 1996, 1994; Vissers et al. 2001; Cyster et al. 2000; Gunn et al. 1998). Within organized lymphoid tissues such as BALT, CXCL13 is predominantly produced by follicular dendritic cells (Fleige et al. 2014; Gunn et al. 1998; Cosgrove et al. 2020). In established allergic airway inflammation, CXCL13 expression is not restricted to these follicular aggregates, but is widely upregulated in the lung, particularly in the airway epithelium (Baay-Guzman et al. 2012). The cellular sources in homeostatic conditions, however, which may participate in the initial steps in these processes before BALT has been formed, are less clear. Some scattered CXCL13-immunoreactive cells have been described in the airway epithelium of non-allergic control mice, but their identity remained undefined (Baay-Guzman et al. 2012).

We here hypothesized that one of the rare epithelial cell types represents this cellular source of CXCL13 in the murine airway epithelium in homeostatic condition, focussing upon neuroendocrine cells. We addressed this question by light and electron microscopic immunohistochemical approaches and in silico analysis of publicly available single cell sequencing data sets.

## Materials and methods

### Animals

Mice were housed under specific pathogen free (SPF) conditions (10 h dark, 14 h light) with free access to food and water. This study was carried out in accordance with the recommendations of the European Communities Council Directive of 24 November 1986 (86/609/EEC). The protocol was approved by the local authorities, i.e., Regierungspräsidium Giessen, Germany (reference no. 572_M and 571_M). Wild-type C57BL/6Rj mice (Janvier Labs, Le Genest-Saint-Isle, France, Cat#5751862) (*n* = 57) and transgenic mice *ChAT-*eGFP (enhanced green fluorescent protein) (B6.Cg-Tg(RP23-268L19-EGFP)2Mik/J, Jackson Laboratory, Bar Harbour, USA, Cat# JAX:007902) (*n* = 7) at least 12 weeks of age and from both genders were used. Mice were killed by inhalation of an overdose of 5% isoflurane (Abbott, Wiesbaden, Germany) and exsanguination through abdominal blood vessels.

### 3R statement

In this study, more than 100 tissue specimens were collected, investigated, and analyzed. In order to adhere to the 3R principle (reduction, replacement, and refinement) in animal experiment principle (Russell and Burch 1992), the number of animals used for this study was kept to a minimum by taking multiple organs (urethra, trachea, thymus, gall bladder, and lung, also used for other projects) from the same animal and by taking specimens from animals that have been sacrificed for other purposes.

### Tissue preparation

For whole mount staining and qualitative RT-PCR, the trachea was removed and opened by cutting the trachealis muscle longitudinally to expose the lining epithelium. The trachea was then pinned on a piece of wax to align the epithelial layer approximately in the same plane. Then, the samples were either used for RT-PCR (*n* = 7) as described later or for whole mount immunostaining (*n* = 19). Samples subjected to whole mount staining were fixed overnight by immersion in Zamboni’s fixative, composed of 2% formaldehyde (Carl Roth) and 15% picric acid (Merck, Darmstadt, Germany), and then washed in 0.1 M phosphate buffer.

Trachea, lung, and spleen processed for sectioning were dissected either freshly or after transcardiac perfusion with Zamboni solution, after initial perfusion with a rinsing solution containing heparin (2 ml/l; 10,000 U; Ratiopharm, Ulm, Germany) and procaine hydrochloride (5 g/l; Merck), pH 7.4. Subsequently, all tissues were immersed in Zamboni’s fixative overnight at 4 °C, washed in 0.1 M phosphate buffer, and, if processed for cryosectioning, stored overnight in 18% sucrose in the same buffer (Merck). Afterward, specimens were embedded in an OCT cryostat sectioning medium (Sakura Finetek, Staufen, Germany) and stored at −20 °C until further processing. Samples of three animals were routinely embedded in paraffin after immersion fixation and buffer wash.

### Precision-cut lung slices

Lungs were taken from 8 to 12-week-old female C57BL/6Rj mice (*n* > 5). Animals were sacrificed as described above, and the airways were filled via the cannulated trachea with 1.5% low melting agarose (Bio-Rad Medical Diagnostics, Dreieich, Germany) dissolved in HEPES-Ringer buffer (4-(2-hydroxyethyl)-1-piperazineethanesulfonic acid (10 mM), KCl (5.6 mM), NaCl (140 mM), MgCl_2_ (1 mM), CaCl_2_ (2.2 mM), glucose (10 mM)). Lungs were removed and transferred to ice-cold HEPES-Ringer buffer to solidify the agarose. The lung lobes were cut into 350-µm-thick slices using a vibratome (VT10000S, Leica, Wetzlar, Germany). Slices were incubated in HEPES-Ringer buffer supplemented with penicillin (100 U/ml, PAA, Etobicoke, Canada) and streptomycin (0.1 mg/ml, PAA) for at least 1.5 h at 37 °C to remove the agarose followed by fixation with Zamboni’s fixative overnight and subsequent immunostaining.

### Immunohistochemistry of tracheal whole mounts and precision-cut lung slices

Tracheal whole mount preparations and precision-cut lung slices were permeabilized with 0.3% Triton X-100 in PBS (0.005 M phosphate buffer with 0.08 M NaCl, pH 7.4) for 2 h; then, non-specific binding sites were saturated by incubation with blocking solution (4% normal horse serum and 1% bovine serum albumin) for 2 h. Primary antibodies (Supplementary table 1) were applied overnight at room temperature. Samples were then incubated for 2 h with blocking solution and incubated overnight with secondary antibodies (Supplementary table 2). Nuclei were labeled with DAPI (1 μg/ml; D9542, Sigma-Aldrich, St. Louis, USA); samples were post-fixed in 4% formaldehyde (Carl Roth, Karlsruhe, Germany) for 10 min and coverslipped in Mowiol (Merck). Whole mounts were evaluated using confocal laser scanning microscopes (LSM 710, Zeiss; FLUOVIEW FV3000, Olympus, Tokyo, Japan; TCS SP8 LIGHTNING, Leica) equipped with appropriate filter and laser sets. Whole trachea overview images were created using the LSM 710 (Zeiss); single pictures were stitched manually using Inkscape version 0.92.4 (https://inkscape.org/de/).

### Immunohistochemistry of sections

For cryosections, 10-μm-thick sections were cut using a microtome (CM-1900 cryostat; Leica). Sections were incubated for 1 h with a blocking solution containing 10% normal horse serum, 0.5% Tween 20, and 0.1% bovine serum albumin. Primary antibodies were applied overnight (see supplementary table 1). After primary antibodies, samples were washed and incubated for 1 h with secondary antibodies (supplementary table 2). Nuclei were labeled with DAPI. Afterwards, samples were post-fixed in 4% paraformaldehyde for 10 min and coverslipped with a drop of carbonate-buffered glycerol (pH 8.6). Immunolabelling for lymphocyte markers was conducted on 7-µm-thick paraffin sections. Sections were deparaffinised, subjected to antigen retrieval by microwave treatment for 10 min in citrate buffer (pH 6.0), and then immunolabelled according to the same protocol as cryosections. The specificity of antibodies was validated by (1) omission of primary antibody to control the specificity of the secondary antibody. (2) For CXCL13 primary antibody, a preabsorption control was done on tracheal cryosections and whole mounts using the immunizing antigen (full-length recombinant CXCL13, 470-BC-025, R&D Systems; 10 μg/ml overnight at room temperature), followed by the described immunostaining protocol. (3) Tissue sections from spleen were used as a positive control for CXCL13 immunolabeling and for antibodies against lymphocyte markers. Sections were evaluated by epifluorescence microscopy (Axioplan 2, Zeiss, Oberkochen, Germany) or a confocal laser scanning microscope (LSM 710, Zeiss) equipped with appropriate filter and laser sets.

### Cell counting

For quantitative assessment of double- or triple-labeling immunofluorescence in whole mount tracheal preparations (PGP9.5/CXCL13 (*n* = 4 for studying cranio-caudal distribution; *n* = 5 for colocalization studies), CGRP/CXCL13 (*n* = 5), or PGP9.5/CXCL13/eGFP (ChAT) (*n* = 6)), Z-stack images through the whole thickness of the epithelium were taken with a 25× water immersion objective lens and a LSM710 confocal laser scanning microscope (Zeiss). In total, 30 image stacks per trachea were obtained by a randomized scheme. The absolute number of immunoreactive cells was quantified manually using ImageJ plug-in cell counter. For each trachea, the total count of single and double positive cells and their percentages and average numbers per mm^2^ were calculated. To quantify immunoreactive epithelial cells in the bronchi, double-labeling immunofluorescence with antibodies against CGRP and CXCL13 was performed. Longitudinal sections of the whole lung including the trachea were analyzed (5 animals, 3–4 sections each with a distance of at least 50 µm from each other). Counting was done manually using an Axioplan 2 epifluorescence microscope equipped with a 40× objective lens, evaluating the entire section plane.

### Electron microscopy

Tracheas were fixed for at least 24 h in 2% paraformaldehyde and 1.5% glutaraldehyde (Merck) in 0.1 M phosphate buffer (pH 7.4). After fixation, specimens were washed in HEPES buffer 0.15 M, pH 7.4 (5 × 10 min), osmicated for 2 h in aqueous 1% osmium tetroxide (Sigma-Aldrich), washed in distilled water, contrasted in 1% uranyl acetate (Merck) overnight, and embedded in epon (Agar Scientific, Essex, UK). Ultrathin sections (80 nm) were cut using an ultramicrotome (Reichert Ultracut E, Leica).

For pre-embedding immuno-electron microscopy, tracheal cryosections of 40-µm thickness were used (CM-3050S cryostat; Leica). Floating sections were rinsed in PBS, and unspecific protein binding sites were saturated with 10% normal porcine serum in 0.005 M PBS for 1 h. Sections were incubated overnight with goat polyclonal antibody against CXCL13 (1:400 AF470, R&D Systems) or rabbit polyclonal antibody against αCGRP (1:20,000; T-4032, Peninsula Laboratories) followed by incubation for 1 h with peroxidase-conjugated pig anti-rabbit Ig (1:100; P0217, Dako, Santa Clara, USA) to detect CGRP or with biotinylated secondary donkey anti-goat IgG (1:400; 705–065-147, Dianova) to detect CXCL13. In the latter case, sections were further incubated for 1 h with peroxidase-coupled streptavidin (1:100; 016–030-084, Dianova). Sections were rinsed in PBS, followed by 0.05 M Tris–HCl buffer (pH 8.6), and peroxidase activity was visualized using a solution containing 15 mg/ml nickel ammonium sulfate (Honeywell, NJ, USA) and 0.125 mg/ml 3.3′-diaminobenzidine-hydrochloride (DAB) (Sigma-Aldrich) in 0.05 M Tris–HCl buffer, for 10 min before adding H_2_O_2_ (Fluka) at a final concentration of 0.0023% for additional 45 min. Sections were washed in Tris–HCl buffer, osmicated for 30 min in aqueous 1% osmium tetroxide (Sigma-Aldrich), and washed in distilled water. Specimens were stained overnight en bloc with 1% uranyl acetate (Merck), dehydrated, and flat-embedded in epon (Agar Scientific). Regions containing immunolabeled cells were selected by light microscopy (Leica ICC50 W), trimmed, and transferred to blank epon blocks for ultrathin sectioning (80 nm). To control the specificity of the secondary antibody, samples were also prepared using the same protocol without a primary antibody. To further validate the specificity of the biotinylated secondary antibodies used in combination with the CXCL13 primary antibody, 10-μm-thick tracheal sections were air-dried for 1 h and unspecific protein binding sites were saturated with 10% normal swine serum in PBS + S (0.005 M phosphate buffer, with 0.15 M NaCl, pH 7.4) for 1 h. Sections were then incubated overnight either with goat polyclonal antibody to CXCL13 (1:400 AF470, R&D Systems) and rabbit polyclonal antibody to αCGRP (1:20,000; T-4032, Peninsula Laboratories) or with rabbit polyclonal antibody to αCGRP only. The sections were incubated with biotinylated donkey anti-goat IgG (1:400; 705–065-147, Dianova) for 1 h followed by incubation for 1 h with Cy3-coupled streptavidin (1:5000; 016–160-084, Dianova) and donkey anti-rabbit Ig Alexa 488 (1:500, A-21206, Invitrogen). Samples were post-fixed in 4% formaldehyde for 10 min and coverslipped with a drop of carbonate-buffered glycerol (pH 8.6) and evaluated by epifluorescence microscopy (Axioplan 2, Zeiss).

Ultrathin sections were analyzed using a transmission electron microscope (EM 902 N, Zeiss, Oberkochen, Germany) equipped with a slow-scan 2 K CCD camera (TRS, Tröndle, Moorenweis, Germany). For vesicle diameter measurements, immunoreactive cells in all analyzed sections were captured with 20,000 magnification (2 CGRP-immunoreactive cells and 5 CXCL13-immunoreactive cells). Only vesicles with clear borders were considered for measurements (in total 69 and 174 vesicles in CGRP- and CXCL13-immunoreactive cells, respectively). Two central perpendicular axes for each vesicle were measured with ImageJ software.

### Flow cytometry

Flow cytometry analyses were performed as described before (Wang et al. 2021). Nine female adult C57BL/6 J mice were sacrificed and immediately transcardially perfused with a solution containing polyvinylpyrrolidon (Roth, 25 g/l) and procaine hydrochloride (5 g/l), followed by perfusion with PBS (Gibco, Darmstadt, Germany). Cranial (cartilage rings 1–3) and caudal (cartilage rings 8–10) ends of the trachea were separately dissected. Samples of three animals were pooled; two independent experiments were performed. Both parts of the trachea were cut into small pieces and digested in RPMI 1640 medium (Gibco) containing collagenase A (1 mg/ml, Roche, Munich, Germany) and DNase I (0.05 mg/ml, Roche) at 37 °C in a shaking water bath. Digested tissue was passed 4–5 times through a 3-ml syringe attached with a 21 G needle and filtered through a 70-µm filter. Cells were centrifuged (350 × *g* for 5 min at 4 °C), and red blood cells were lysed by adding red blood cell lysis buffer (Qiagen Germany). The cells were resuspended in FACS buffer (PBS containing 2% fetal calf serum (Gibco) and 2 mM EDTA (Sigma-Aldrich)), incubated with Fc blocker (1:10 dilution, Miltenyi Biotec, Bergisch Gladbach, Germany) for 10 min, followed by staining with antibodies (Supplementary table 3) for 30 min at 4 °C. Subsequently, cells were washed and dissolved in FACS buffer. Flow cytometry analysis was performed using a MACSQuant Analyzer 10 flow cytometer (Miltenyi Biotec); data were analyzed with FlowJo (Version 10.7.; Tree Star, Ashland, USA). Dead cells were excluded by staining with Zombie NIR™ Fixable Viability dye (dilution 1:1000, BioLegend, catalogue #423105).

### Reverse transcription PCR and quantitative PCR (RT-PCR, RT-qPCR)

For qualitative analyses, the tracheal epithelial layer of adult C57BL/6 J mice (*n* = 7) was abraded using cotton swabs soaked with RLT buffer (Qiagen, Hilden, Germany) supplemented with 1% β-mercaptoethanol (Sigma). Total RNA from abraded tracheal epithelium was isolated by using the RNeasy Mini kit (Qiagen, Hilden, Germany) according to the manufacturer’s instructions. For cDNA synthesis, 8 μl RNA was incubated with 1 µl 10× DNase reaction buffer and 1 µl DNase (1 U/μl; Invitrogen) for 15 min at 25 °C to degrade contaminating DNA. To each sample, 1 μl of EDTA (25 mM) was added. After 10-min incubation at 65 °C, samples were rapidly cooled for 2 min and 9 μl reaction mixture of 1 μl oligo-dT (50 μM), 1 μl dNTPs (10 mM), 1 μl Superscript RNase H-RT (200 U/μl), 4 μl 5× first-strand buffer, and 2 μl dithiothreitol (0.1 M) was added to each sample. All reagents were from Invitrogen, except dNTPs which were from Qiagen. PCR was performed with cDNA samples using primers (specified below) for CGRP, CXCL13, and β-actin (housekeeping gene). The following protocol was used: 1 μl cDNA as template, 2 μl MgCl_2_ (25 mM), 2.5 μl 10× PCR buffer II, 0.75 μl dNTPs (10 mM), 0.75 μl of each primer (20 pM), 0.25 μl AmpliTaq Gold DNA polymerase (5 U/μl; all reagents were purchased from Thermo Fisher), and 17.75 μl H_2_O. PCR was conducted with the following temperature and time profile: 95 °C for 12 min for initial denaturation, followed by 39 cycles at 95 °C for 20 s, 60 °C for 20 s, and 72 °C for 20 s and a final extension at 72 °C for 7 min. β-Actin was used as an efficacy control for PCR, and omission of the reverse transcriptase during cDNA synthesis served as a negative control. Water negative controls were also processed with each reaction to test for the absence of genomic DNA contamination. The PCR products were subjected to electrophoresis in ethidium bromide-containing 2% agarose gels. A 100-bp DNA ladder (Invitrogen) was run as a marker, and bands were detected by UV light. Primer pairs used: CGRP fwd: atgcagatgaaagccaggga, rev: aagttgtccttcaccacacc, product length 158 bp, GeneBank accession number NM001289444; CXCL13 fwd: aggccacggtattctggaag, rev: agcttggggagttgaagaca, product length 250 bp, NM018866; and β-actin fwd: gtgggaatgggtcagaagg, rev: ggcatacagggacagcaca, product length 300 bp, NM007393.

For RT-qPCR, samples from cranial (cartilages 1–3) and caudal (cartilages 8–10) trachea were taken from 6 animals and processed for further analysis. Total RNA was isolated by using the RNeasy Micro kit (Qiagen) according to the manufacturer’s instruction. Contaminating DNA was removed by on column digest using the RNase-Free DNase set (Qiagen). Reverse transcription was done as described for RT-PCR. iQ SYBR Green Supermix (Bio-Rad) was used for qPCR with the following temperature and time profile: 95 °C for 5 min for initial denaturation, followed by 40 cycles at 95 °C for 20 s, 60 °C for 20 s, and 72 °C for 20 s. β-Actin served as a housekeeping gene. Samples processed without reverse transcriptions and water controls were included to control for absence of DNA contamination. Gel electrophoresis was done as described for RT-PCR. Primer pairs used: CD22 fwd: gcgctttccagagagtgaca, rev: ccgcttctgtatcaccgagt, product length 112 bp, GeneBank accession number NM001043317; CD19 fwd: gaggcacgtgaaggtcattg, rev: gaagaatctcctggcgggg, product length 192 bp, GeneBank accession number NM009844; CD4 fwd: agtagttcaagtggtggccc, rev: gagcccaaggaaacccagaa, product length 172 bp, GeneBank accession number NM013488; CD8 fwd: ggacgaagctgactgtggtt, rev: ggtggtaaggctgcatgtca, product length 137 bp, GeneBank accession number NM009858. β-Actin and CXCL13 primers were the same as described for RT-PCR. Relative expression was calculated by 2^−ΔCt^, where ΔCt was calculated as the Ct of the gene of interest minus the Ct of the housekeeping gene.

### In silico analysis of published mRNA sequencing data

Previously published gene expression data (Plasschaert et al. 2018, data set GSE102580, and Montoro et al. 2018, data set GSE103354) of murine tracheal epithelial cells from uninjured C57Bl6/J mice were downloaded from SPRING and re-analyzed. Analysis and re-clustering were done using the Seurat R package (version 2.3.4) (Wolock et al. 2019). Principle component analysis (PCA) was done, and UMAP (Uniform Manifold Approximation and Projection) was used for non-linear dimensional reduction (Satija et al. 2015). Cells were represented in a two-dimensional UMAP plot, and clusters were identified and annotated based on the composition of typical marker genes. From data set GSE102580, heat maps were created using ggplots function heatmap.2 in R package (https://cran.r-project.org/). Sequencing analysis was performed by two independent operators resulting in the same outcome. The top 200 most expressed genes in neuroendocrine cells were selected. About 60 of them were differentially expressed (fold change > 1) compared to other cell types. From these ~ 60 genes, a heat map was created from CXCL13^+^ and CXCL13^−^ neuroendocrine cells. Another analysis was done by analyzing the original data set again using different selection criteria (fold change > 2.5, average TPM value more than 1 in either CXCL13 positive or negative groups or both), and these genes were used to create further heat maps.

### Statistical analysis

Data are presented as individual data points with mean ± standard error of the mean (SEM). Statistical analyses of data were performed with GraphPad Prism software version 7 (La Jolla, CA, USA). Data were analyzed by non-parametric tests (Mann–Whitney *U* test) or chi-squared test. Differences were considered statistically significant when *p* ≤ 0.05.

## Results

### Scattered tracheal epithelial cells express CXCL13

**Fig. 1:**
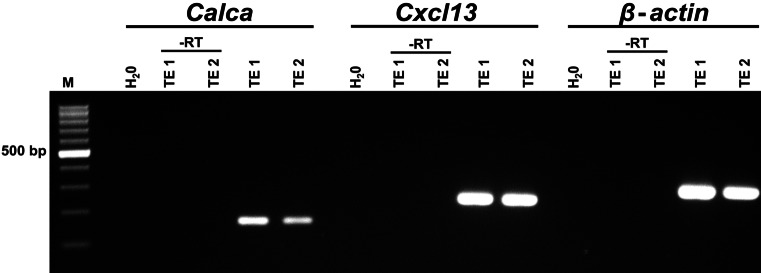
*Cxcl13*- and *Calca*-mRNAs are present in the murine tracheal epithelium. RT-PCR experiments with cDNA obtained from tracheal epithelium of two C57BL/6RJ animals using primers for *Cxcl13* (250 bp), *Calca* (158 bp), and *β-actin* (300 bp). Amplicons of *Calca*, *Cxcl13*, and *β-actin* are detected in both tracheal epithelium samples (TE). Controls = RNA samples processed without reverse transcriptase (-RT) and water (H_2_O) without adding cDNA

RT-PCR revealed expression of CXCL13 in the abraded tracheal epithelium (Fig. 1). This approach was sensitive enough to detect mRNAs selectively expressed by rare cell types, such as *Calca-*mRNA, encoding the precursor of the neuropeptide CGRP, which is restricted to neuroendocrine cells in the tracheal epithelium (Fig. 1).

**Fig. 2:**
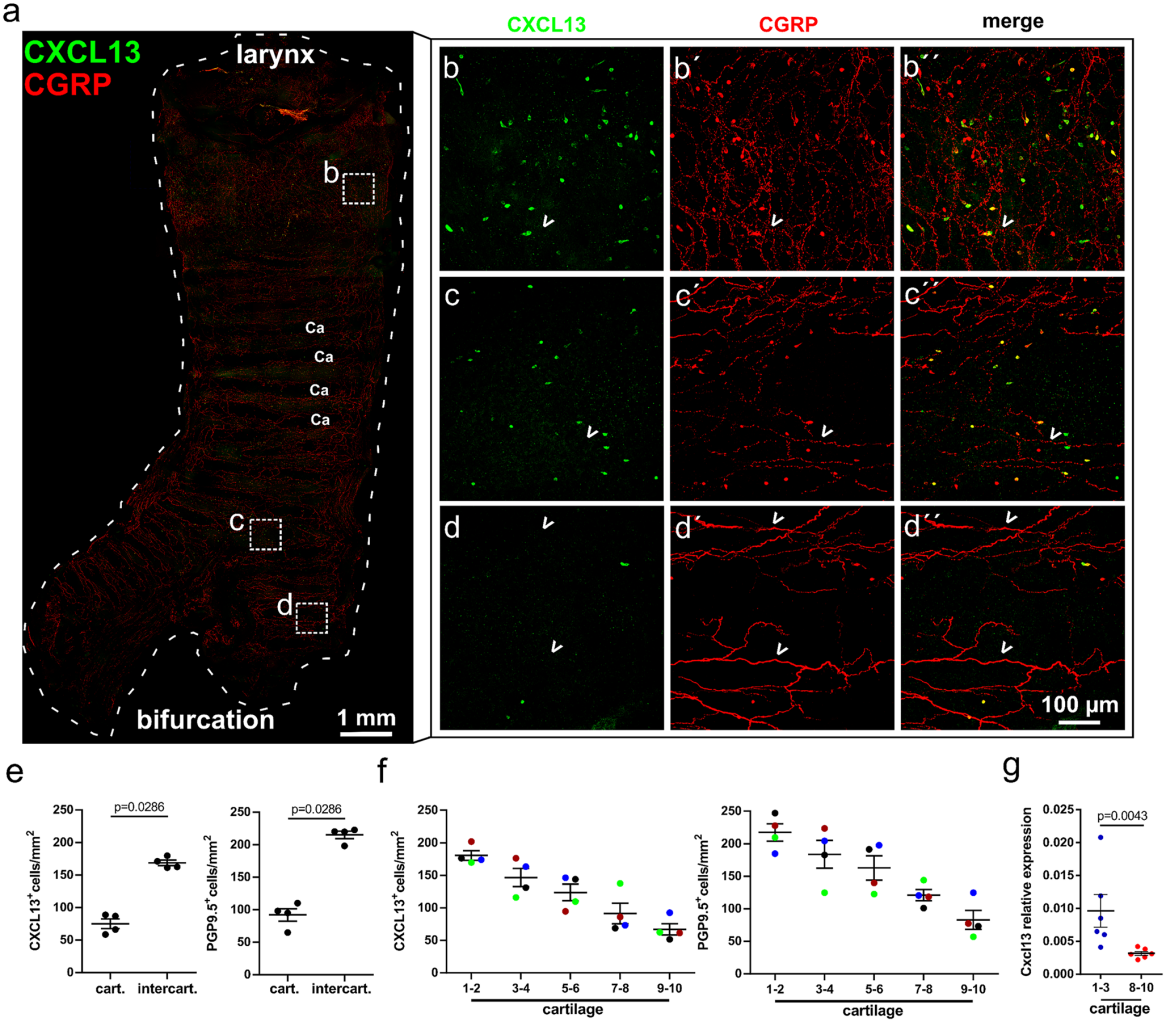
CXCL13-positive and neuroendocrine cells are unevenly distributed in the trachea. **a**–**d** Tracheal whole mount immunohistochemistry, CLSM, with antibodies against CXCL13 (green) and CGRP (red), labeling single neuroendocrine cells and nerve fibers. The density of neuroendocrine (CGRP-positive) cells, CXCL13-immunoreactive cells, and nerve fibers is higher between the cartilage rings (Ca). The density of neuroendocrine cells and CXCL13-immunoreactive cells decreases from cranial (larynx) (**b**) to caudal (bifurcation) (**d**); each channel is individually shown in Supplementary Fig. 1. Numerous CGRP-positive nerve fibers ( <) are visible throughout the whole trachea. Maximum intensity projection of z-stack of confocal optical sections. **e, f** Cell densities (mean ± SEM) quantified on tracheal whole mounts double-labelled for CXCL13 and PGP9.5 (neuroendocrine cell marker). Immunoreactive cells dominate in intercartilage regions (**e**), and their density continuously declines along the cranio-caudal axis (**f**). In **f**, counts include both the area overlying a cartilage and the next intercartilage region; colour coding along the cranio-caudal axis identifies data from the same trachea. **g** RT-qPCR. *Cxcl13* expression is about 3 times higher in tracheal rings 1–3 compared to rings 8–10

Immunolabeling localized CXCL13 immunoreactivity to solitary cells in the tracheal epithelium (Fig. 2a–d and Supplementary Fig. 1). The specificity of this labeling was validated by appropriate positive (spleen; Supplementary Fig. 2a) and negative controls. CXCL13-immunoreactive cells were absent both after preabsorption of the primary antibody with the immunizing peptide (full-length recombinant CXCL13 protein; Supplementary Fig. 2b, c, and d) and when the primary antibody was omitted to test for specificity of secondary antibodies (Supplementary Fig. 2a). The density of CXCL13-immunoreactive cells was highest in the intercartilage regions (approximately doubled compared to regions overlying cartilage, Fig. 2e) and decreased by more than 50% from cranial (larynx) to caudal (bifurcation) (Fig. 2f). Only few cells were visible below the bifurcation region (Fig. 2a, d and Supplementary Fig. 1). Real-time PCR confirmed the cranio-caudal difference, demonstrating threefold higher expression of *Cxcl13*-mRNA in the cranial 3 than in the caudal 3 tracheal ring segments (Fig. 2g).

B cells, the major target cells of CXCL13, were observed within both the epithelial layer and the lamina propria (Supplementary Fig. 3a), but at rare occurrence with up to only 3 cells per longitudinal section of the trachea. T cells appeared slightly more frequent, although also being rare (Supplementary Fig. 3a). Flow cytometry also revealed low numbers of lymphocytes in trachea (Supplementary Fig. 3b-d). In contrast to CXCL13, a cranio-caudal difference in B and T cells was neither noted in flow cytometry (Supplementary Fig. 3b-d), nor in relative expression of B cell (CD19, CD22) and T cell markers (CD4, CD8) (Supplementary Fig. 4).

### CXCL13 is restricted to neuroendocrine cells in the tracheal epithelium

**Fig. 3:**
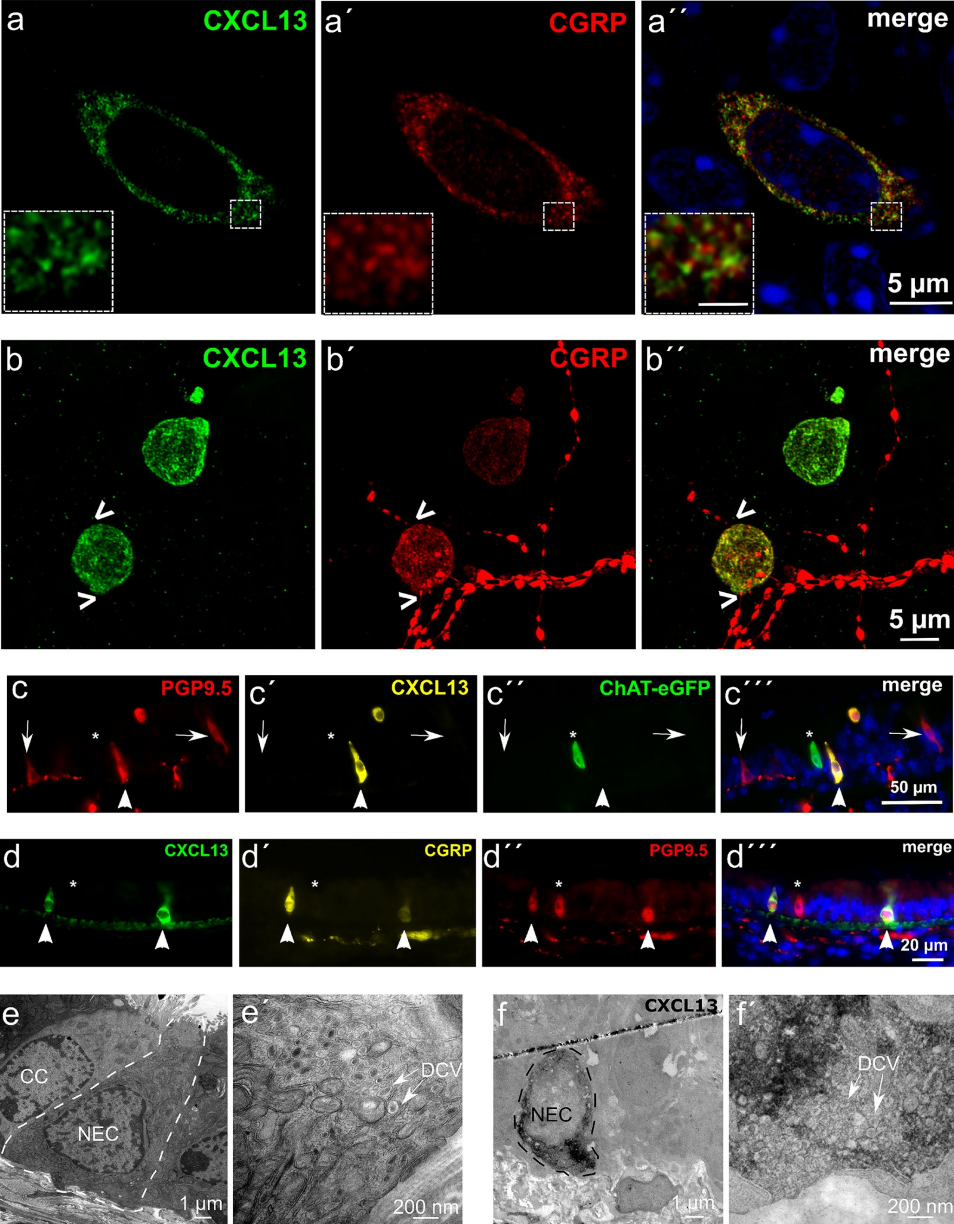
CXCL13 is restricted to neuroendocrine cells in the tracheal epithelium. **a**, **b** High-resolution of tracheal whole mount immunohistochemistry, labeling CXCL13^+^ cells in green and CGRP^+^ cells and nerve fibers in red, revealed CXCL13 and CGRP colocalization within the same cell. Inset in **a** shows the magnified region of the labeled cell with only limited overlap of immunoreactivites (scale bar 1 µm). Images were acquired using a confocal laser scanning microscope (FLUOVIEW FV3000; Olympus), single confocal optical section. **b** CXCL13^+^ cell in contact to a CGRP^+^ nerve fiber ( <). Maximum intensity projection of z-stack of confocal optical sections. **c** Immunohistochemistry of a tracheal cryosection from a ChAT-eGFP animal. In the tracheal epithelium, single cells are double-stained with antibodies against PGP9.5 and CXCL13 (arrowhead) or PGP9.5 only (arrow). An eGFP-positive cell (asterisk) is not labeled with antibodies against CXCL13 or PGP9.5. PGP9.5^+^ nerve fibers are in contact to PGP9.5^+^ epithelial cells. **d** Triple-immunofluorescence of tracheal cryosection shows co-labeling of single epithelial cells for CXCL13 (green), CGRP (yellow), and PGP9.5 (red) (arrowheads). A single PGP9.5-labeled cell with neither CXCL13 nor CGRP immunolabeling is also present (asterisk). **e–f** Transmission electron microscopy. **e** Ultrastructure of a tracheal neuroendocrine cell (NEC) with a pyramidal or flask-like shape and a small apical part reaching the lumen with microvilli. **e′** Higher magnification of the basal part, showing the presence of numerous dense core vesicles (DCV). **f** Ultrastructural immunohistochemistry with antibodies against CXCL13 shows an immunoreactive cell with the diffuse DAB reaction product. **f′** Higher magnification of the basal part, showing the presence of numerous DCV

Neuroendocrine cells, characterized by immunoreactivity to PGP9.5, showed the same distribution pattern as CXCL13-immunoreactive cells with preferential occurrence overlying ligaments between cartilage rings (Fig. 2e) and marked cranio-caudal gradient (Fig. 2f). Double-labeling revealed colocalization of immunoreactivities to CXCL13 and CGRP, a peptide of neuroendocrine cells, within the same cell (Fig. 2a–d, Supplementary Fig. 1), but with only limited overlap of immunofluorescence signals at high-resolution confocal microscopy (Fig. 3a). CGRP is also contained in peptidergic sensory nerve fibers ramifying underneath and within the tracheal epithelium (Figs. 2a–d and 3b, Supplementary Fig. 1 and Supplementary Video 1) (Krasteva et al. 2011; Terada et al. 1992; Kusindarta et al. 2004). Such nerve fibers made contact to some CXCL13^+^/CGRP^+^ epithelial cells, but themselves were CXCL13-negative (Fig. 3b and Supplementary Video 1). Likewise, CXCL13 immunoreactivity colocalized with PGP9.5, another neuroendocrine cell marker, in solitary epithelial cells, while PGP9.5-immunoreactive nerve fibers were CXCL13-negative (Fig. 3c, d).

Pre-embedding immuno-electron microscopy finally validated the neuroendocrine identity of CXCL13-immunoreactive cells. It shall be emphasized that this does not allow to define the precise subcellular localization of CXCL13, since DAB is a diffusible reaction product that can precipitate microns away from the site of enzymatic generation. Ultrastructurally, solitary neuroendocrine cells are characterized by basal aggregations of small (80–100 nm in diameter) granules with variable densities (dense core vesicles) (Ericson et al. 1972) (Fig. 3e). Both CGRP- and CXCL13-immunoreactive cells invariably showed these accumulations of dense core vesicles (Fig. 3f, Supplementary Fig. 2e, and controls shown in Supplementary Fig. 2f, g and h). Their average short and long central axis diameters were 75 and 90 nm, respectively, in CGRP-immunoreactive cells (69 vesicles, *n* = 2 cells), and 76 and 91 nm in CXCL13-immunoreactive cells (174 vesicles, *n* = 5 cells).

In contrast, solitary cholinergic chemosensory cells were devoid of CXCL13 immunoreactivity, as revealed by double-labeling experiments utilizing a TRPM5 antibody as a chemosensory cell marker (Supplementary Fig. 5a) and by triple-labeling experiments of tracheas from ChAT-eGFP reporter mice with PGP9.5 and GFP antibodies (Fig. 3c, Supplementary Fig. 5b and c).

### Two-thirds of the tracheal neuroendocrine cell population exhibit the CXCL13^+^ phenotype

**Fig. 4:**
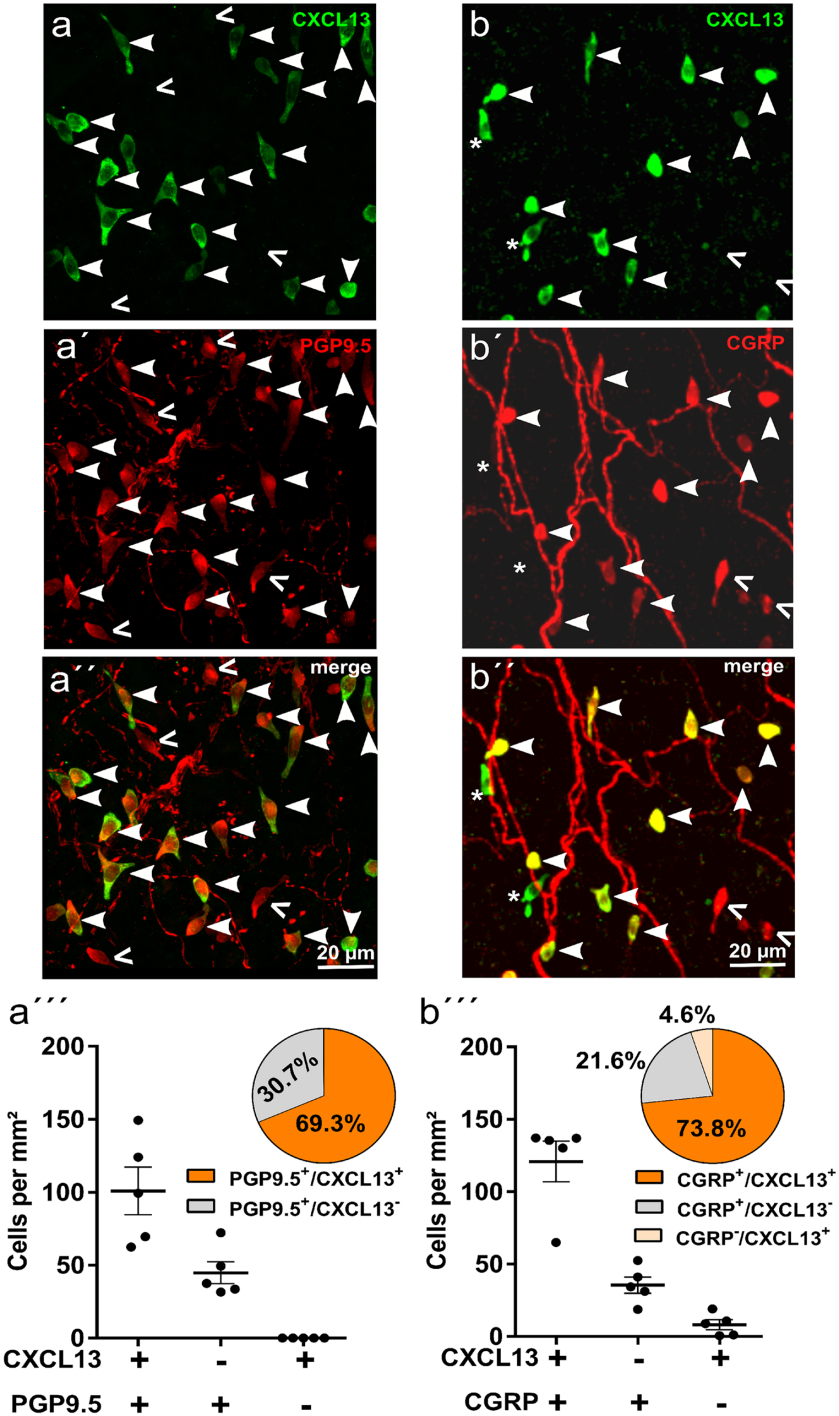
CXCL13 is expressed in two-thirds of the murine tracheal neuroendocrine cells. **a**, **b** Immunohistochemistry of tracheal whole mounts and the corresponding quantification of their immunoreactive cells; maximum intensity projections of z-stacks of confocal optical sections. **a** Immunohistochemistry with antibodies against CXCL13 (**a**) (green) and PGP9.5 (**a′**) (red), labeling single neuroendocrine cells and nerve fibers. CXCL13^+^/PGP9.5^+^ cells are indicated by arrowheads; CXCL13^−^/PGP9.5^+^ cells are indicated by ( <). Data points in the scatter plot (**a‴**) represent mean values of counts in one trachea (*n* = 5 tracheas); mean and SEM are indicated. The pie chart shows the percentages of CXCL13^+^/PGP9.5^+^ and CXCL13^−^/PGP9.5^+^ cells (*n* = 2254 cells pooled from 5 tracheas). **b** Immunohistochemistry with antibodies against CXCL13 (**b**) (green) and CGRP (**b′**) (red), labeling single neuroendocrine cells and nerve fibers. CXCL13^+^/CGRP^+^ cells are indicated by arrowheads; CXCL13^−^/CGRP^+^ cells are indicated by ( <); CXCL13^+^/CGRP^−^ cells are indicated by (*). Data points in the scatter plot (**b‴**) represent mean values of counts in one trachea (*n* = 5 tracheas); mean and SEM are indicated. The pie chart shows the percentages of phenotypes (*n* = 2650 cells pooled from 5 tracheas)

While all CXCL13^+^ cells were PGP9.5^+^, there was a population of PGP9.5^+^ (neuroendocrine) cells not labeled by the CXCL13 antibody (Fig. 4a). Relative frequencies of immunoreactive phenotypes and their densities in the tracheal epithelium were quantified in whole mount preparations. Double-positive cells (CXCL13^+^/PGP9.5^+^) made up the majority (69%) of neuroendocrine cells and CXCL13^−^/PGP9.5^+^ cells accounted for 31% (Fig. 4a‴). Using CGRP antibody as a marker for neuroendocrine cells in double-labeling experiments revealed highly similar numbers and frequencies (73% double-positive, 22% CXCL13^−^/CGRP^+^), albeit a small fraction (5%) of CXCL13^+^ cells without CGRP immunoreactivity appeared in these experiments (Fig. 4b and Supplementary Fig. 6).

**Fig. 5:**
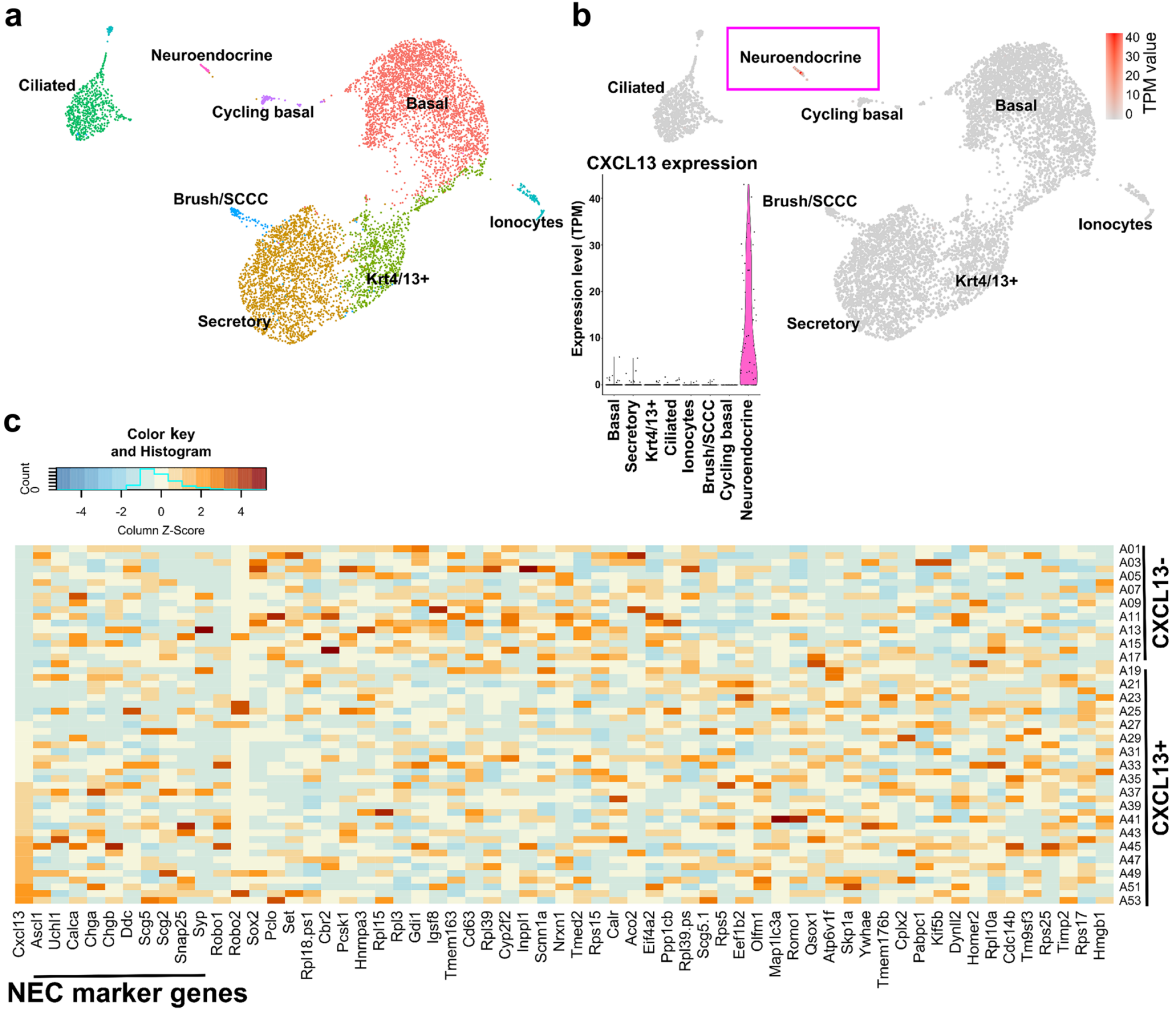
In silico analysis of single-cell mRNA sequencing data revealed CXCL13 expression predominantly in neuroendocrine cells of the tracheal epithelium. **a**–**c** In silico analysis of published sequencing data (GSE102580) of murine tracheal epithelial cells (Plasschaert et al. 2018). **a** SPRING plot (Uniform Manifold Approximation and Projection, UMAP) shows eight distinct cell clusters, namely basal, secretory, Krt4/13^+^, ciliated, solitary cholinergic chemosensory (brush/tuft), cycling basal and solitary neuroendocrine cells, and ionocytes. **b** SPRING and violin plots showing that *Cxcl13*-mRNA is predominantly expressed within the neuroendocrine cell cluster. **c** Heat map shows the most differentially expressed genes (fold change > 1) between CXCL13^+^ and CXCL13^−^ neuroendocrine cells among the 200 highest expressed genes within the neuroendocrine cell cluster and typical neuroendocrine cell marker genes (e.g., *Ascl1*, *Uchl1*, *Calca*, *Chga*, *Chgb*)

These immunohistochemical findings were validated and supplemented with in silico analysis of published sequencing data of murine tracheal epithelial cells (Plasschaert et al. 2018; Montoro et al. 2018). We were able to reproduce the clustering reported by the sequencing laboratories, identifying eight distinct cell clusters, namely basal, secretory, Krt4/13^+^, ciliated, ionocytes, solitary cholinergic chemosensory (brush/tuft), cycling basal, and solitary neuroendocrine cells in the data set GSE102580 from Plasschaert and coworkers (Fig. 5a), and seven distinct cell clusters, namely basal, club, ciliated, goblet, solitary cholinergic chemosensory (brush/tuft), solitary neuroendocrine cells, and ionocytes in the data set GSE103354 from Montoro and coworkers (Supplementary Fig. 7a). This analysis revealed that neuroendocrine cells are the major source of *Cxcl13*-mRNA in the mouse tracheal epithelium, whereas its expression was negligible (expressed by 1% of cells or less) in other epithelial cell types. *Cxcl13*-mRNA was found in 68% (36/53; data set GSE102580; Fig. 5b) and 79% (54/68; data set GSE103354; Supplementary Fig. 7b, c), respectively, matching the 69–73% determined by immunohistochemical double labeling.

Further analysis revealed a panel of ~ 100 genes that showed a different expression level (fold change > 2.5) between *Cxcl13*^+^ and *Cxcl13*^*−*^ cells. While we could not identify a gene known to be involved in CXCL13 signaling pathways, *Robo1* was found among these differentially regulated genes, being upregulated in the CXCL13^+^ cell population (15/36 versus 4/17 in CXCL13^−^ cells) (Supplementary Fig. 8a and b). *Robo* expressed by lung neuroendocrine cells is required to cluster into neuroepithelial bodies (Branchfield et al. 2016). Unbiased analysis of both data sets, however, allowed no subclustering of neuroendocrine cells based on distinct gene expression patterns (Supplementary Fig. 7d and 8c). Specifically, we did not find differences between CXCL13^+^ and CXCL13^−^ neuroendocrine cells with respect to the highest expressed genes, including commonly used neuroendocrine cell marker genes (e.g., *Ascl1*, *Uchl1*, *Calca*, *Chga*, *Chgb*), analyzed in data set GSE102580 (Fig. 5c).

### CXCL13 is less expressed in broncho-pulmonary solitary and clustered neuroendocrine cells

In intrapulmonary airways, the vast majority (95.3%; 1475/1548 cells from 5 animals) of all CGRP^+^ cells was aggregated to neuroepithelial bodies and only 4.7% of all CGRP^+^ cells were solitary neuroendocrine cells (73/1548) (Supplementary Fig. 9a).

**Fig. 6:**
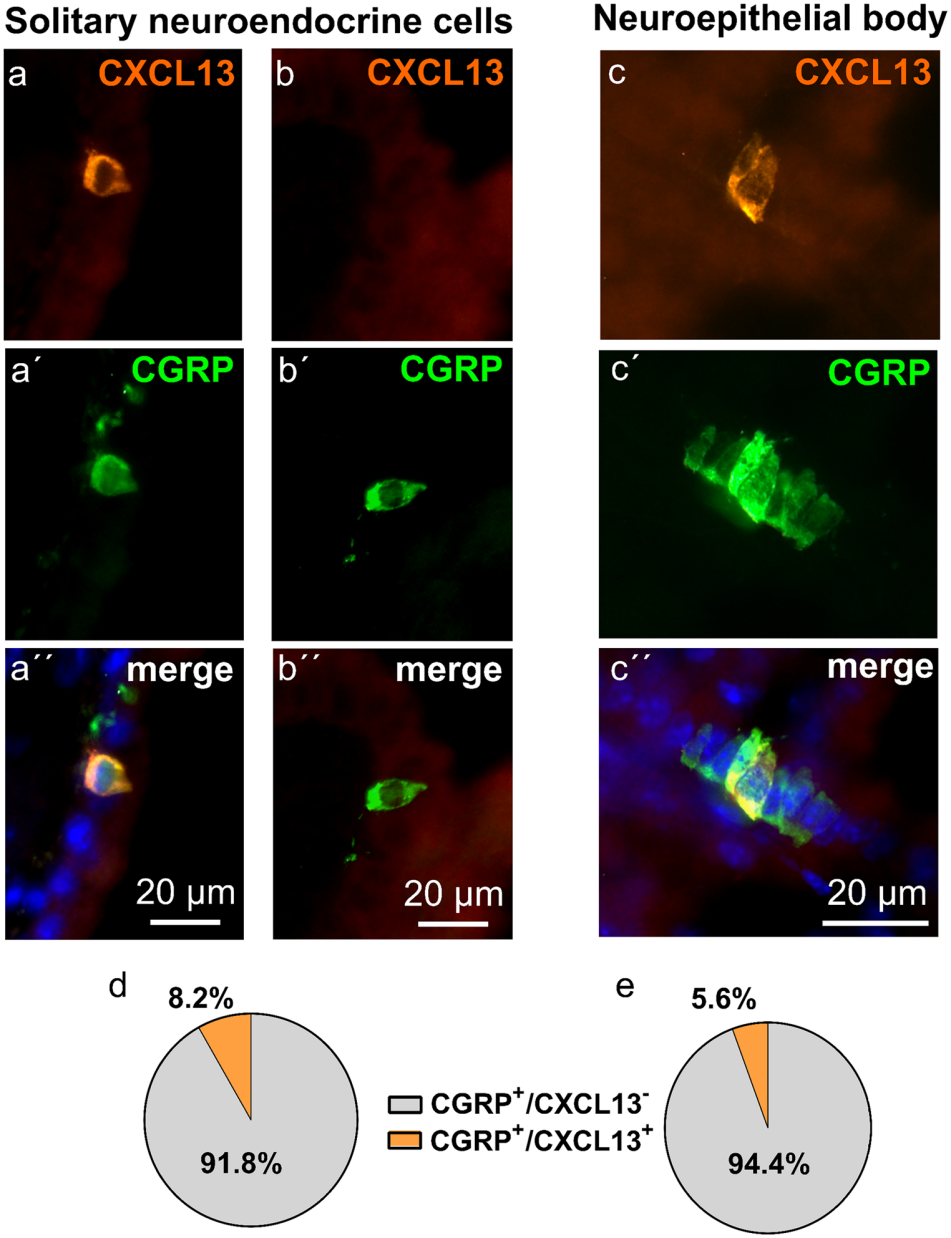
CXCL13 is less expressed in murine broncho-pulmonary solitary and clustered neuroendocrine cells. Immunohistochemistry of lung cryosections with antibodies against CXCL13 (orange) and CGRP (green) and the relative frequencies of immunoreactive phenotypes. **a** Solitary neuroendocrine cell co-labeled with antibodies against CXCL13 and CGRP. **b** Solitary neuroendocrine cell only labeled with antibodies against CGRP. **c** A cluster of neuroendocrine cells (neuroepithelial body) consisting of more than 7 cells, 2 of them are co-labeled with antibodies against CXCL13 and CGRP. **d** Pie chart shows percentages of CXCL13^+^/CGRP^+^ and CXCL13^−^/CGRP^+^-immunolabeled cells in solitary neuroendocrine cells (*n* = 73 cells pooled from 5 animals). **e** Pie chart shows the percentage of CXCL13^+^/CGRP^+^ and CXCL13^−^/CGRP^+^-immunolabeled cells in neuroepithelial bodies (*n* = 1475 cells pooled from 5 animals)

In double-labeled tissue sections, CXCL13 immunoreactivity was observed both in solitary and in clustered CGRP^+^ neuroendocrine cells, albeit at much lower frequencies than in the trachea (Fig. 6, Supplementary Figs. 9 and 10). Double-positive cells (CXCL13^+^/CGRP^+^) accounted for 8% (6/73 cells) of solitary intrapulmonary neuroendocrine cells (Fig. 6d) and for 6% (82/1475 cells) of CGRP-immunoreactive cells in neuroepithelial bodies (Fig. 6e). CXCL13^+^/CGRP^−^ epithelial cells were not observed.

## Discussion

Our data add a homeostatic chemokine, CXCL13, to the portfolio of messengers produced by neuroendocrine cells. As their name reflects, secretory products identified so far included biogenic amines, e.g., serotonin and GABA, which are typically used as neuronal signaling molecules, and peptides also known from neurons or from endocrine cells, e.g., CGRP. A basal accumulation of dense core vesicles is the ultrastructural hallmark of airway neuroendocrine cells, and such amines and peptides are often co-stored in and co-released from such vesicles (Fujita et al. 1988). The limited overlap of CXCL13 and CGRP immunoreactivities which we noted in confocal microscopy, however, may result from an independent secretory pathway for CXCL13, matching their anticipated mode of function. CGRP is a pre-formed mediator released by exocytosis of the storage vesicles upon acute stimulation, e.g., exposure of human airway neuroendocrine cells to volatile chemicals (Gu et al. 2014). Chemokines are preferentially released through constitutive secretory pathways (Morales-Tirado et al. 2004; Reikvam et al. 2019). The specific secretory pathways of CXCL13 yet have not been investigated, but its homeostatic role in governing B cell migration through tissues is compatible with a constitutive mode of secretion. On the other hand, the marked cranio-caudal gradient in numbers of CXCL13 expressing tracheal neuroendocrine cells was not paralleled with a similar uneven distribution of B cells along the cranio-caudal axis. Thus, pure numbers of CXCL13 expressing cells alone are not sufficient to induce regional inhomogeneity in B cell distribution in the trachea under steady-state conditions.

Double-labeling experiments with antibodies against two different neuroendocrine cell markers and in silico analysis of available single cell RNA sequencing data very consistently revealed that approximately two-thirds of tracheal neuroendocrine cells express CXCL13, while a third is CXCL13-negative at a given time point. Despite this heterogeneity, however, an unbiased scRNA-seq analysis of overall gene expression data did not yield subclusters of neuroendocrine cells, and expression of the most characteristic and defining genes of neuroendocrine cells was not different between CXCL13^+^ and CXCL13^−^ cells. Hence, we consider CXCL13^+^ and CXCL13^−^ as phenotypes of one cell type rather than proposing to subdivide neuroendocrine cells into two distinct entities defined by the expression or not of CXCL13.

This concept implies plasticity of CXCL13 expression in neuroendocrine cells, as it is also a characteristic feature of several other cell types. CXCL13 expression can be induced in various airway epithelial cells, pneumocytes, macrophages, and pulmonary reticular cells/fibroblasts in diverse inflammatory conditions such as allergic airway inflammation, environmental pollutant-induced lung cancer, and bacterial and viral infection (Baay-Guzman et al. 2012; Wang et al. 2015; Bracke et al. 2013; Denton et al. 2019; Frija-Masson et al. 2017). In contrast to neuroendocrine cells, however, there is no or only negligible steady state or homeostatic expression of CXCL13 in these cells. Notably, the raw single-cell RNA sequencing data we analyzed were generated in entirely independent laboratories (Montoro et al. 2018; Plasschaert et al. 2018), showing that baseline expression of CXCL13 in tracheal neuroendocrine cells does not reflect an inflammatory condition that may have occurred unperceived in one particular animal house. Known inducers of CXCL13 expression in non-lymphoid tissue under inflammatory settings include type I interferon, IL-1, IL-17, IL-22, and tumor necrosis factor α (Frija-Masson et al. 2017; Kuroda et al. 2016; Neyt et al. 2016; Barone et al. 2015; Bénézech et al. 2015; Rangel-Moreno et al. 2011). The driver of CXCL13 expression in tracheal neuroendocrine cells under baseline conditions remains to be determined.

In contrast to the trachea, CXCL13 expression occurred in only less than 10% of broncho-pulmonary neuroendocrine cells. During development, pulmonary neuroendocrine cells differentiate as solitary cells, presumably from basal cells, and then aggregate to neuroepithelial bodies through directed migration within the epithelial cell layer (Kuo and Krasnow 2015; Noguchi et al. 2015). It is unlikely that this clustering induced downregulation of CXCL13 expression compared to the trachea, because solitary pulmonary neuroendocrine cells exhibited equally low frequency of CXCL13 expression. Hence, less bronchopulmonary than tracheal neuroendocrine cells express CXCL13, regardless whether they are aggregated into neuroepithelial bodies or lie solitarily in the epithelium. One possible cause of this difference might be that tracheal and bronchopulmonary neuroendocrine cells represent two distinct entities with profound functional differences. Single-cell sequencing data allowing direct comparison between tracheal and intrapulmonary neuroendocrine cells to address this question are not available yet. Alternatively, neuroendocrine cells of the trachea and intrapulmonary airways basically represent the same cell type at different anatomical locations, but are exposed to different environmental cues driving CXCL13 expression preferentially in the trachea. The particularly high frequency of neuroendocrine cells in the cranial, i.e., sublaryngeal part of the trachea, is suggestive of a sentinel function towards inhaled substances, in line with the observation of the expression of olfactory receptors in cultured human solitary tracheobronchial neuroendocrine cells (Gu et al. 2014). Higher exposure to such air-borne stimuli might promote CXCL13 expression in the proximal airways. Yet, this model is not supported by our observation that the relative frequency of CXCL13^+^ cells changed abruptly from trachea to lung but not gradually along the trachea. Lastly, the cellular microenvironment of tracheal and bronchopulmonary neuroendocrine cells differs, and tracheal neuroendocrine cells might be instructed to express CXCL13 by specific cells of their vicinity. One candidate might be cholinergic chemosensory cells which share two peculiar characteristics of spatial distribution with tracheal neuroendocrine cells: enrichment in epithelial areas between cartilages and a striking cranio-caudal gradient in cell frequency. Direct evidence of cross-talk between these rare cell types, however, has not been reported yet.

## Supplementary information

**Supplementary Table 1** Primary antibodies for immunohistochemistry.

**Supplementary Table 2** Secondary antibodies for immunohistochemistry.

**Supplementary Table 3** Antibodies for flow cytometry.

**Supplementary Fig. 1** Individual channels of the tracheal whole-mount immunostaining depicted in Fig. 2a, CLSM, with antibodies against CXCL13 (green) and CGRP (red), labeling single neuroendocrine cells and nerve fibers. The merged image to the left is the same as depicted in Fig. 2a; here, individual channels are shown in addition. Maximum intensity projection of z-stack of confocal optical sections.

**Supplementary Fig. 2 Control experiments validating immunostaining. (a)** Immunohistochemistry of spleen cryosections, showing CXCL13-immunoreactive cells, probably follicular dendritic cells, in the white pulp (left image). No staining could be overserved when the primary antibody (CXCL13) was omitted (right picture). **(b)** Immunohistochemistry of tracheal cryosection. Control experiment (left) using antibodies against CXCL13, showing labeling of single cells, whereas such cells are missing after preabsorption of the primary antibody with the immunizing peptide (full length recombinant CXCL13 protein) (right image). **(c and d)** Immunohistochemistry of tracheal whole mounts; maximum intensity projections of z-stacks of confocal optical sections. Positive control experiments (upper panels) using antibodies against PGP9.5 (c) or CGRP (d) to identify neuroendocrine cells, and labeling with an antibody against CXCL13 showing double- and single-positive cells. Preabsorption with the immunizing peptide (full length recombinant CXCL13 protein) completely blocked CXCL13-immunoreactivity (lower panels), whilst immunoreactivity to PGP9.5 or CGRP was not affected. **(e–g)** Control experiments corresponding to immuno-electron microscopy shown in Fig. 3f. **(e)** Ultrastructural immunohistochemistry with antibodies against CGRP shows an immunoreactive flask-shaped cell with DAB reaction product, the lower panel is a higher magnification of the basal part, showing the presence of numerous DCV. **(f)** Omission of the primary CGRP antibody, no labeling visible in a neuroendocrine cell (NEC) identified by the presence of dense core vesicles (DCV) in the basal cell portion (lower panel, higher magnification of boxed region in the upper panel). **(g)** Omission of the primary CXCL13 antibody, no labeling visible in a neuroendocrine cell (NEC) identified by the presence of dense core vesicles (DCV) in the basal cell portion (lower panel, higher magnification of boxed region in the upper panel). **(h)** Control experiment for the experiments shown in Fig. 3f, in which biotinylated secondary antibodies were used, to exclude unspecific labeling of endogenous biotin within the neuroendocrine cells. CGRP was used as a marker to identify neuroendocrine cells. The left panel shows labeling of neuroendocrine cells with antibodies against CXCL13 and CGRP (arrowheads). Right panel, omission of primary antibody (CXCL13), no unspecific labeling of the neuroendocrine cell (CGRP^+^) (arrowheads) caused by the biotinylated secondary antibody is visible, unspecific labeling is detected in other cell types (asterisks).

**Supplementary Fig. 3 Lymphocytes in the trachea.** (a) Triple-labelling for B cells (B220), T cells (CD3) and CXCL13, 7 µm paraffin sections, epifluorescence microscopy; spleen served as positive control (upper row) and as control for secondary reagents by omitting primary antibodies (lower row). In the trachea, rarely occurring B cells were observed in the epithelial layer (open arrowhead) and in the lamina propria (arrowhead). Arrows: CXCL13-positive epithelial cells; asterisks: CD3-positive T cells in the lamina propria. Merged images also include DAPI stining for nuclei. **(b-d)** Flow cytometry. (b) Gating strategy. (c, d) Numbers of hematopoetic cells in general (CD45^+^) and of B cells (CD19^+^) in cranial (cartilage rings 1–3) and caudal (cartilage rings 8–10) trachea. Each data point represents the value from 3 pooled samples. There is no decrease in numbers in cranio-caudal direction.

**Supplementary Fig. 4 Expression of B and T cell markers in cranial and caudal trachea, RT-qPCR. (a, b)** Agarose gel electrophoresis of amplicons obtained with primers for B cell (a) and T cell (b) markers; β-actin served as efficacy control. 1–3: tracheal rings 1–3, 8–10: tracheal rings 8–10, H_2_O: water control, M: 100 bp marker, -RT: samples processed without reverse transcription. **(c, d)** Neither B cell markers (CD19, CD22) nor T cell markers (CD4, CD8) are higher expressed in cranial (tracheal rings 1–3) than in caudal (tracheal rings 8–10) trachea. Reference gene: β-actin. *Cxcl13* expression of the same samples is depicted in Fig. 2g.

**Supplementary Fig. 5 Tracheal whole mount staining with markers for solitary cholinergic chemosensory cells and CXCL13 revealed no colocalization. (a)** Immunohistochemistry of tracheal whole mount of a C57BL/6Rj mouse. TRPM5-immunoreactive cells (solitary cholinergic chemosensory cells) are not labeled with antibodies against CXCL13. **(b)** Triple-labeling immunofluorescence of a tracheal whole mount from a ChAT-eGFP animal, GFP-immunoreactive cells (magenta) (cholinergic chemosensory cells) are not labeled with antibodies against CXCL13 (green). CXCL13-immunoreactive cells are also labeled with antibodies against PGP9.5 (red). Maximum intensity projections of z-stacks of confocal optical sections in (a) and (b). **(c)** Percentages of immunoreactive phenotypes determined from preparations as depicted in b. Data points in the scatter plot represent mean values of counts in one trachea (n = 5 tracheas); mean and SEM are indicated. CXCL13-positive cells are predominantly also labeled with antibodies against PGP9.5 (~ 67% of all evaluated cells). Only ~ 1% of all evaluated cells were single CXCL13-positive. No cells were found showing co-labeling with antibodies against CXCL13 and GFP.

**Supplementary Fig. 6** Percentages of colocalization of CXCL13 with either PGP9.5 or CGRP are comparable (69.3% and 73.3%; p = 0.9358; Chi-square test; n = 5 whole mounts, mean ± SEM).

**Supplementary Fig. 7 In silico-analysis of single cell mRNA sequencing data of tracheal neuroendocrine cells, resource data set GSE103354. (a)** SPRING plot (Uniform Manifold Approximation and Projection, UMAP) shows seven distinct cell clusters, namely basal, club, ciliated, goblet, solitary cholinergic chemosensory (brush/tuft), solitary neuroendocrine cells and ionocytes. **(b and c)** SPRING and violin plots showing that *Cxcl13*-mRNA is predominantly expressed within the neuroendocrine cell cluster. **(d)** Uniform Manifold Approximation and Projection (UMAP), each dot represents a single neuroendocrine cell (n = 68). There is no subclustering of neuroendocrine cells based on distinct gene expression patterns.

**Supplementary Fig. 8 In silico-analysis of single cell mRNA sequencing data of tracheal neuroendocrine cells revealed no subclustering. (a and b)** Heat maps showing differences in gene expression levels (fold change > 2.5) between CXCL13^+^ and CXCL13^−^ neuroendocrine cells. Upper heat map **(a)**: genes upregulated in CXCL13^+^ cells. Lower heat map **(b)**: genes downregulated in CXCL13^+^ cells. **(c)** Uniform Manifold Approximation and Projection (UMAP), each dot represents a single neuroendocrine cell (n = 53). There is no subclustering of neuroendocrine cells based on distinct gene expression patterns. Resource: data set GSE102580.

**Supplementary Fig. 9 CXCL13 expression in tracheal and broncho-pulmonary neuroendocrine cells,** supplementing experimental data depicted in Fig. 6. **(a)** CXCL13 expression within the bronchial epithelium. Left pie chart showing the percentages of CGRP-immunolabeled NEC in lung cryosections (total number of immunoreactive cells = 1548 from 5 animals) organized in NEB or appearing as solitary cells. Scatter plot showing numbers of immunolabeled cells per 2 mm basement membrane counted in tracheal cryosections labeled with antibodies against CGRP and CXCL13. Data points represent mean values of counts in one trachea (n = 5 tracheas); mean and SEM are indicated. Percentages of immunoreactive phenotypes depicted in the right pie chart (n = 413 cells). **(b)** Confocal laser scanning microscopy (Zeiss, LSM 710) of a precision-cut lung slice, double-labeled with CXCL13 and CGRP antibodies; maximum intensity projection of z-stack of confocal optical sections. Boxed areas #1 to #3 are depicted at higher magnification in the lower panels. NEB (boxed areas #1 and #2), a solitary neuroendocrine cell (boxed area #3) and nerve fibers (arrows) are stained with antibodies against CGRP. A CGRP^+^ cell in NEB #1 is co-labeled with antibodies against CXCL13, whereas CGRP^+^ cells of NEB #2 and the solitary neuroendocrine cell are not CXCL13-immunoreactive.

**Supplementary Fig. 10 Neuroendocrine cell phenotypes based on CXCL13 expression within the tracheal and broncho-pulmonary epithelium.** In the tracheal epithelium, two types of solitary neuroendocrine cells (SNEC) are present, CXCL13 pos. or CXCL13 neg. ones (1). In the bronchial epithelium, four types of neuroendocrine cells are present, CXCL13 pos. and CXCL13 neg. solitary neuroendocrine cells (2), and neuroendocrine cells (NEC) which cluster together as NEB (neuroepithelial bodies), consisting of CXCL13 neg. and CXCl13 pos. cells, or only CXCL13 neg. cells (3).

**Supplementary Video 1** Tracheal whole mount staining, 3D-reconstruction of a z-stack of confocal optical sections, with two CXCL13- (green) and CGRP-positive (magenta) cells together with CGRP-imunoreactive nerve fibres. The double-positive cell in the centre comes into direct contact to a CGRP-immunoreactive nerve fibre. Nuclei labelled with DAPI (blue).

## Supplementary Information

Below is the link to the electronic supplementary material.

Supplementary file1 (PDF 132532 KB)

Supplementary file2 (MP4 26234 KB)
