# Supplementary material for: CXCL13 is expressed in a subpopulation of neuroendocrine cells in the murine trachea and lung
Source: Cell Tissue Res. 2021 Nov 11;390(1):35–49. doi: 10.1007/s00441-021-03552-2 (PMC9525416; doi:10.1007/s00441-021-03552-2)
Supplement: Supplementary file 1 — Supplementary file1 (PDF 132532 KB) [file 441_2021_3552_MOESM1_ESM.pdf]

Supplementary Information to

CXCL13 is expressed in a subpopulation of neuroendocrine cells in the murine trachea and lung

Wafaa Mahmoud[1, 2*], Alexander Perniss[1*], Krupali Poharkar[1], Aichurek Soultanova[1], Uwe Pfeil[1], Andreas Hoek[3], Sudhanshu Bhushan[4], Torsten Hain[5], Ulrich Gärtner[1] and Wolfgang Kummer[1]

[1]Institute for Anatomy and Cell Biology, German Center for Lung Research, Excellence Cluster Cardio-Pulmonary Institute (CPI), Justus Liebig University Giessen, 35392, Germany

[2]Department of Anatomy, Faculty of Medicine, Jordan University of Science and Technology, Irbid, Jordan.

[3]Institute for Bioinformatics and Systems Biology, Justus Liebig University Giessen, 35392, Germany

[4]Institute of Anatomy and Cell Biology, Unit of Reproductive Biology, Justus Liebig University Giessen, 35392, Germany

[5]Institute of Medical Microbiology, German Center for Infection Research, Partner Site Giessen-Marburg-Langen, Justus Liebig University Giessen, 35392, Germany

*contributed equally

Corresponding Author: Wolfgang Kummer

Institute for Anatomy and Cell Biology

Justus Liebig University Giessen

Aulweg 123

35392 Giessen

Germany

e-mail: wolfgang.kummer@anatomie.med.uni-giessen.de

Tel.: +49641 47000 / Fax: +49641 47009

Supplementary Table 1. Primary antibodies for immunohistochemistry.

Antigen	Host	Dilution	Company	Catalog #
PGP9.5	rabbit	1:4,000	GeneTex, Irvine, USA	GTX109637
UCHL1 (PGP9.5) (only used for triple labeling in cryosections)	chicken	1:4,000	Novus Biologicals, Littleton, USA	NB110-58872
CXCL13	goat	1:400-800	R&D Systems, Minneapolis, USA	AF470
αCGRP	rabbit	1:20,000	Peninsula Laboratories, San Carlos, USA	T-4032
GFP	chicken	1:4,000	Novus Biologicals,	NB100-161
beta-tubulin IV	mouse	1:1,200	BioGentex, San Ramon, USA	AM178-10M
CD3	rabbit	1:800	abcam	Ab5690
B220 (CD45R)	rat	1:800	Invitrogen	14-0452-82
TPRM5	rabbit	1:4,000	Kaske et al. (2)	

Kaske, S., Krasteva, G., Koenig, P., Kummer, W., Hofmann, T., Gudermann, T., and Chubanov, V. (2007). TRPM5, a taste-signaling transient receptor potential ion-channel, is a ubiquitous signaling component in chemosensory cells. BMC Neurosci. 8, 49.

Supplementary Table 2. Secondary antibodies for immunohistochemistry.

Antigen	Host	Conjugate	Dilution	Company	Catalog #
rabbit Ig	donkey	Cy3	1:2,000	Chemicon, Limburg, Germany	AP182C
goat Ig	donkey	Alexa 488	1:1,000	Molecular Probes, Eugene, USA	A-11055
chicken IgY	donkey	FITC	1:400	Dianova	703-095-155
rabbit Ig	donkey	Cy5	1:400	Dianova	711-175-152
mouse Ig	donkey	Cy3	1:1,000	Dianova	715-165-151
goat Ig	donkey	Cy3	1:1,600	Merck	AP180C
rabbit Ig	donkey	Alexa 488	1:500	Invitrogen, Carlsbad, USA	A-21206
rat Ig	donkey	Cy3	1:1,000	Dianova	712-165-153
chicken IgY	donkey	Cy3	1:2,000	Dianova	703-166-155

Supplementary Table 3. Antibodies for flow cytometry.

Antigen	Host	Conjugate	Dilution	Company	Catalog #
CD45	rat	PE	1:200	Biolegend	103108
CD19	rat	PE/Cy7	1:80	Biolegend	115520
rat IgG2a, κ	rat	PE/Cy7	1:80	Biolegend	400522

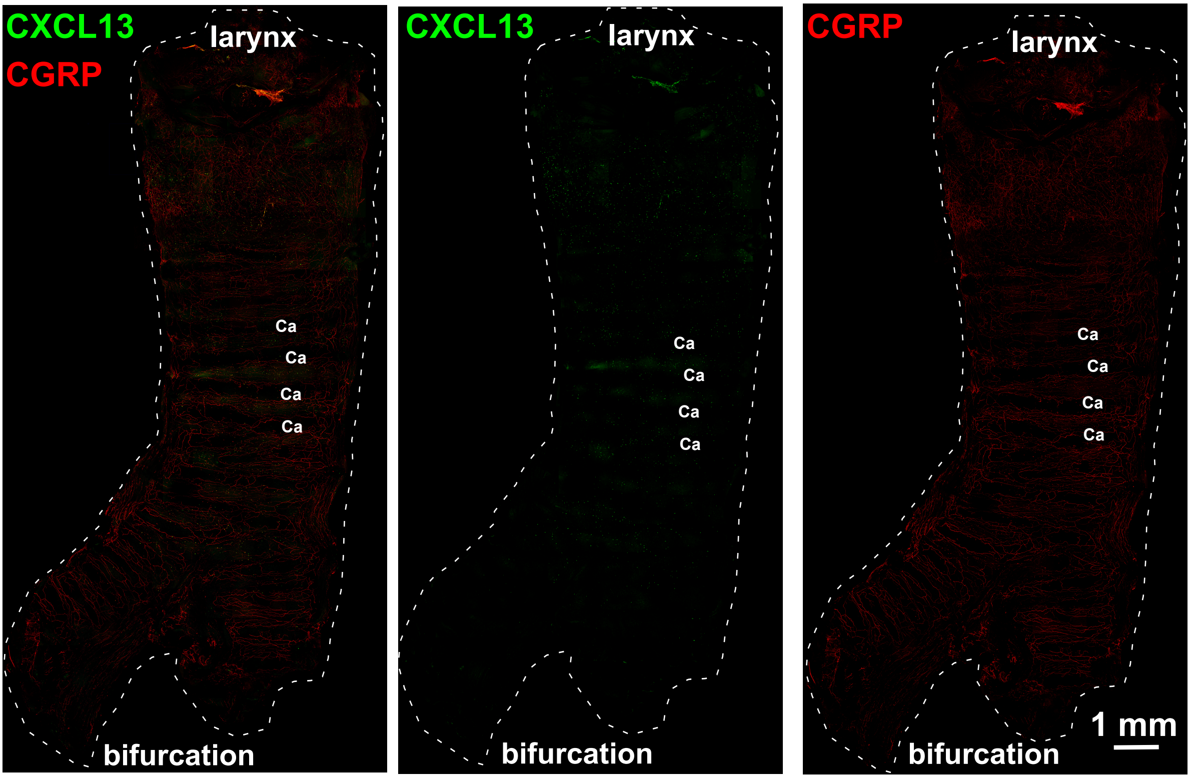

Supplementary Fig. 1 Individual channels of the tracheal whole-mount immunostaining depicted in Fig. 2a, CLSM, with antibodies against CXCL13 (green) and CGRP (red), labeling single neuroendocrine cells and nerve fibers. The merged image to the left is the same as depicted in Fig. 2a; here, individual channels are shown in addition. Maximum intensity projection of z-stack of confocal optical sections.

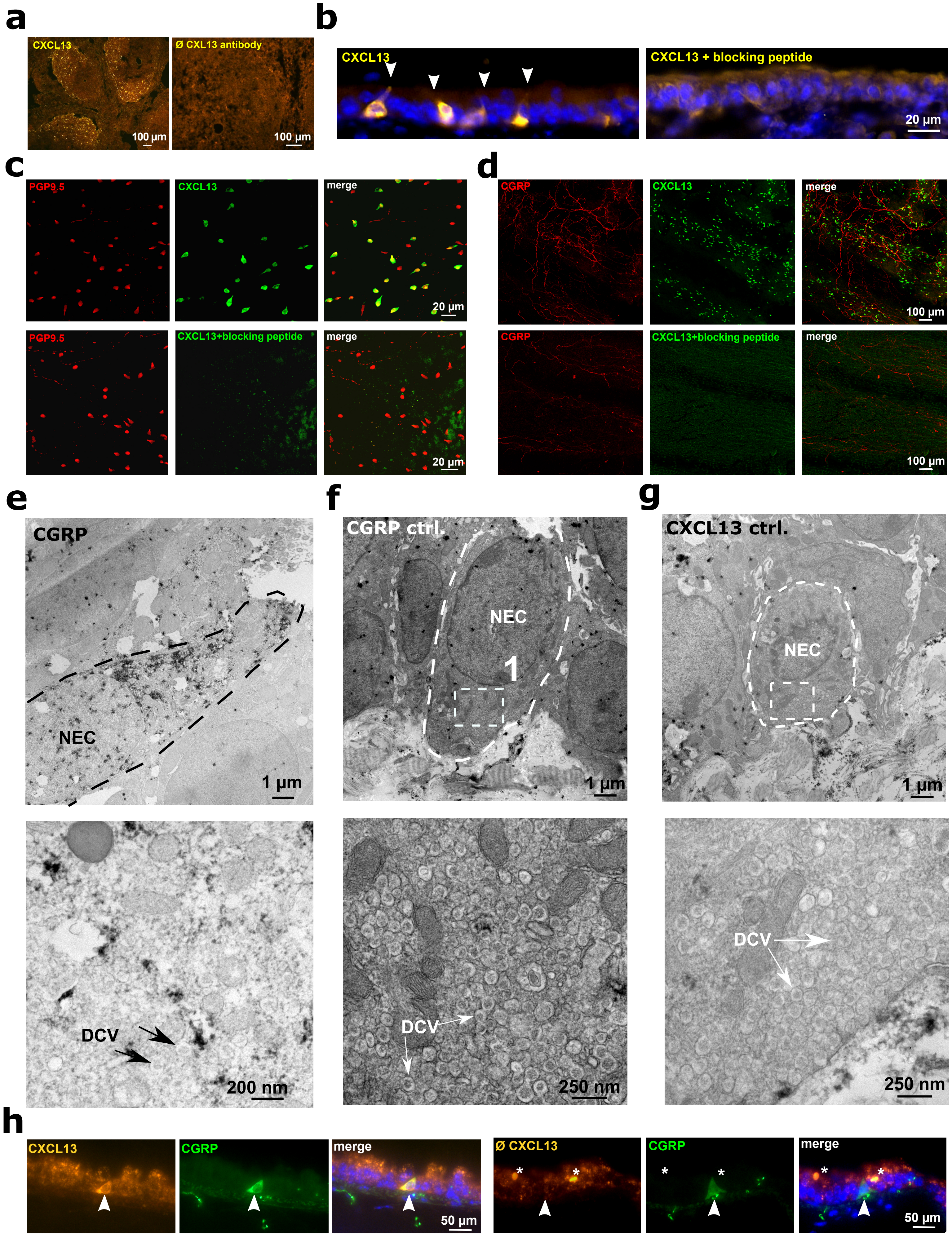
a
CXCL13
100 µm
Ø CXL13 antibody
100 µm
b
CXCL13
CXCL13 + blocking peptide
20 µm
c
PGP9.5
CXCL13
merge
20 µm
PGP9.5
CXCL13+blocking peptide
merge
20 µm
d
CGRP
CXCL13
merge
100 µm
CGRP
CXCL13+blocking peptide
merge
100 µm
e
CGRP
NEC
1 µm
DCV
200 nm
f
CGRP ctrl.
NEC
1
1 µm
DCV
250 nm
g
CXCL13 ctrl.
NEC
1 µm
DCV
250 nm
h
CXCL13
CGRP
merge
50 µm
Ø CXCL13
CGRP
merge
50 µm

Supplementary Fig. 2 Control experiments validating immunostaining. (a) Immunohistochemistry of spleen cryosections, showing CXCL13-immunoreactive cells, probably follicular dendritic cells, in the white pulp (left image). No staining could be overserved when the primary antibody (CXCL13) was omitted (right picture). **(b)** Immunohistochemistry of tracheal cryosection. Control experiment (left) using antibodies against CXCL13, showing labeling of single cells, whereas such cells are missing after preabsorption of the primary antibody with the immunizing peptide (full length recombinant CXCL13 protein) (right image). **(c and d)** Immunohistochemistry of tracheal whole mounts; maximum intensity projections of z-stacks of confocal optical sections. Positive control experiments (upper panels) using antibodies against PGP9.5 (c) or CGRP (d) to identify neuroendocrine cells, and labeling with an antibody against CXCL13 showing double- and single-positive cells. Preabsorption with the immunizing peptide (full length recombinant CXCL13 protein) completely blocked CXCL13-immunoreactivity (lower panels), whilst immunoreactivity to PGP9.5 or CGRP was not affected. **(e-g)** Control experiments corresponding to immuno-electron microscopy shown in Fig. 3f. **(e)** Ultrastructural immunohistochemistry with antibodies against CGRP shows an immunoreactive flask-shaped cell with DAB reaction product, the lower panel is a higher magnification of the basal part, showing the presence of numerous DCV. **(f)** Omission of the primary CGRP antibody, no labeling visible in a neuroendocrine cell (NEC) identified by the presence of dense core vesicles (DCV) in the basal cell portion (lower panel, higher magnification of boxed region in the upper panel). **(g)** Omission of the primary CXCL13 antibody, no labeling visible in a neuroendocrine cell (NEC) identified by the presence of dense core vesicles (DCV) in the basal cell portion (lower panel, higher magnification of boxed region in the upper panel). **(h)** Control experiment for the experiments shown in Fig. 3f, in which biotinylated secondary antibodies were used, to exclude unspecific labeling of endogenous biotin within the neuroendocrine cells. CGRP was used as a marker to identify neuroendocrine cells. The left panel shows labeling of neuroendocrine cells with antibodies against CXCL13 and CGRP (arrowheads). Right panel, omission of primary antibody (CXCL13), no unspecific labeling of the neuroendocrine cell ($CGRP^+$) (arrowheads) caused by the biotinylated secondary antibody is visible, unspecific labeling is detected in other cell types (asterisks).

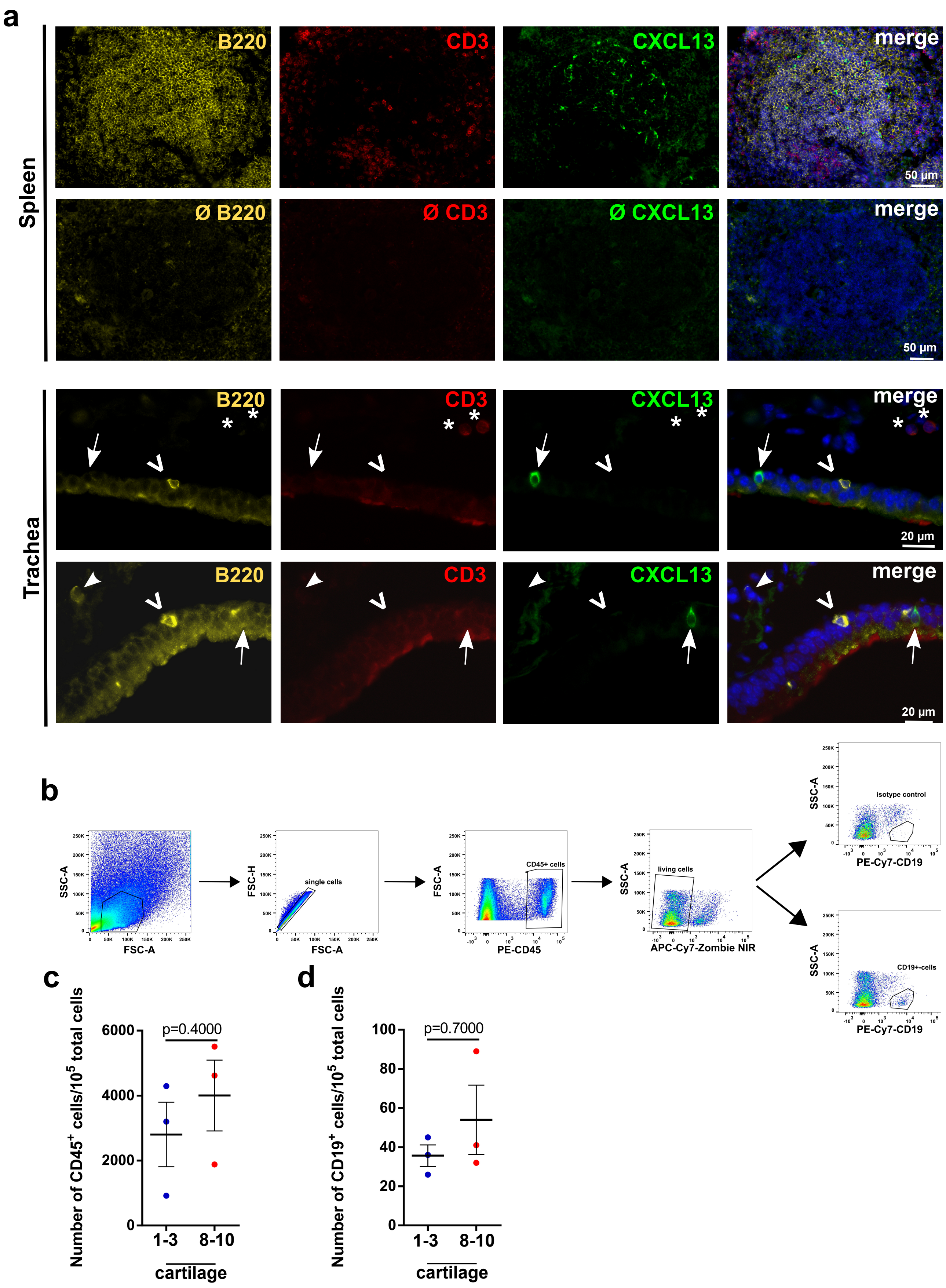

Supplementary Fig. 3 Lymphocytes in the trachea. (a) Triple-labelling for B cells (B220), T cells (CD3) and CXCL13, 7 µm paraffin sections, epifluorescence microscopy; spleen served as positive control (upper row) and as control for secondary reagents by omitting primary antibodies (lower row). In the trachea, rarely occurring B cells were observed in the epithelial layer (open arrowhead) and in the lamina propria (arrowhead). Arrows: CXCL13-positive epithelial cells; asterisks: CD3-positive T cells in the lamina propria. Merged images also include DAPI stining for nuclei. **(b-d)** Flow cytometry. (b) Gating strategy. (c, d) Numbers of hematopoetic cells in general ($CD45^+$) and of B cells ($CD19^+$) in cranial (cartilage rings 1-3) and caudal (cartilage rings 8-10) trachea. Each data point represents the value from 3 pooled samples. There is no decrease in numbers in cranio-caudal direction.

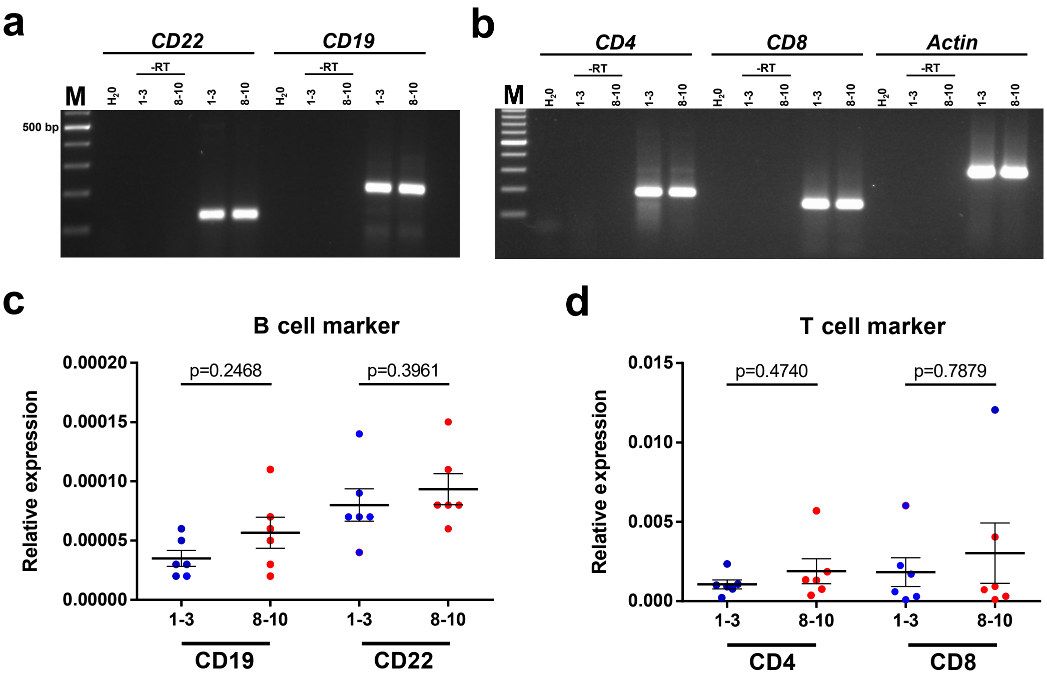

Supplementary Fig. 4 Expression of B and T cell markers in cranial and caudal trachea, RT-qPCR. (a, b) Agarose gel electrophoresis of amplicons obtained with primers for B cell (a) and T cell (b) markers; β-actin served as efficacy control. 1-3: tracheal rings 1-3, 8-10: tracheal rings 8-10, H_2O: water control, M: 100 bp marker, -RT: samples processed without reverse transcription. **(c, d)** Neither B cell markers (CD19, CD22) nor T cell markers (CD4, CD8) are higher expressed in cranial (tracheal rings 1-3) than in caudal (tracheal rings 8-10) trachea. Reference gene: β-actin. *Cxcl13* expression of the same samples is depicted in Fig. 2g.

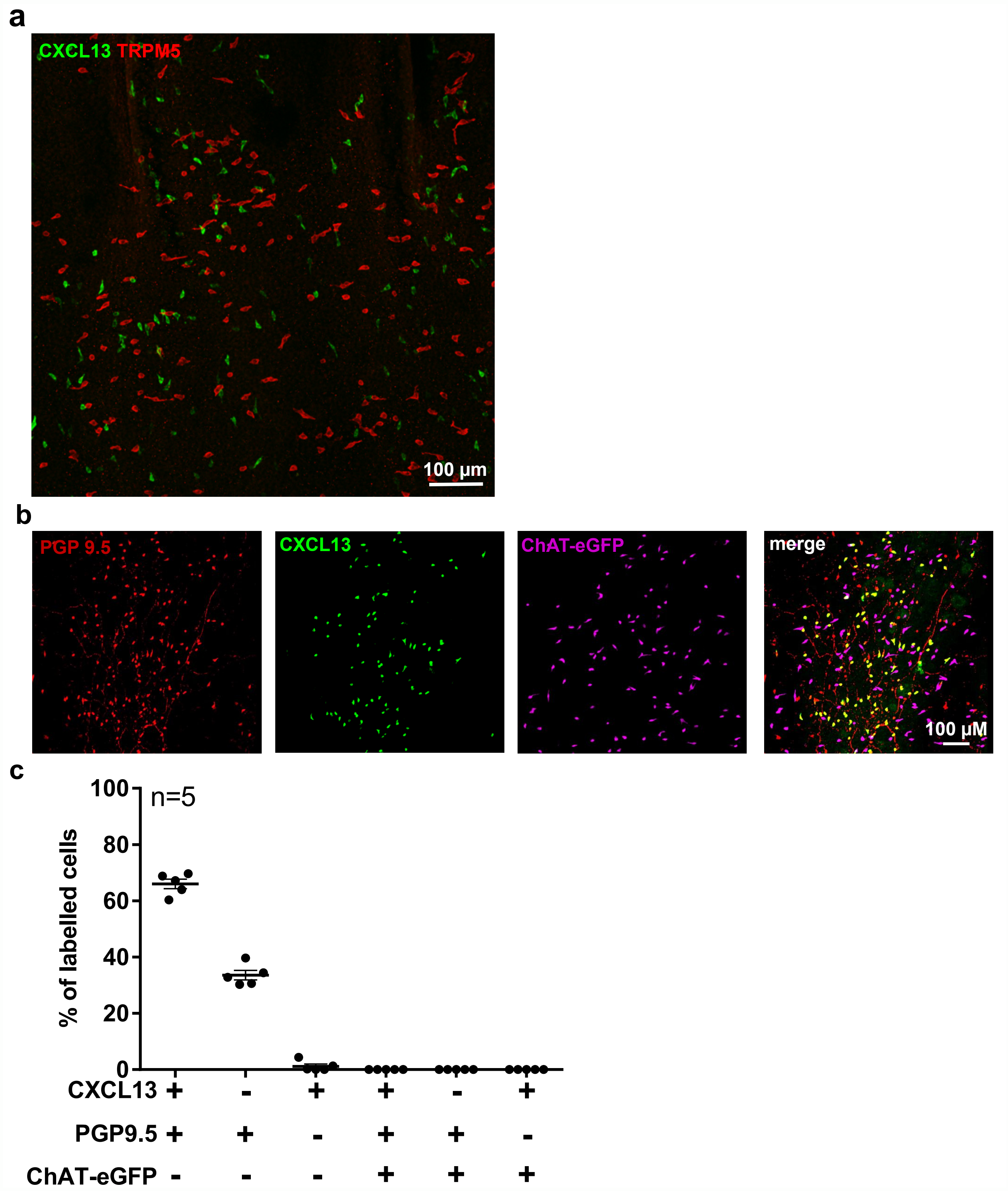

Supplementary Fig. 5 Tracheal whole mount staining with markers for solitary cholinergic chemosensory cells and CXCL13 revealed no colocalization. (a) Immunohistochemistry of tracheal whole mount of a C57BL/6Rj mouse. TRPM5-immunoreactive cells (solitary cholinergic chemosensory cells) are not labeled with antibodies against CXCL13. **(b)** Triple-labeling immunofluorescence of a tracheal whole mount from a ChAT-eGFP animal, GFP-immunoreactive cells (magenta) (cholinergic chemosensory cells) are not labeled with antibodies against CXCL13 (green). CXCL13-immunoreactive cells are also labeled with antibodies against PGP9.5 (red). Maximum intensity projections of z-stacks of confocal optical sections in (a) and (b). **(c)** Percentages of immunoreactive phenotypes determined from preparations as depicted in b. Data points in the scatter plot represent mean values of counts in one trachea (n=5 tracheas); mean and SEM are indicated. CXCL13-positive cells are predominantly also labeled with antibodies against PGP9.5 (~67% of all evaluated cells). Only ~1% of all evaluated cells were single CXCL13-positive. No cells were found showing co-labeling with antibodies against CXCL13 and GFP.

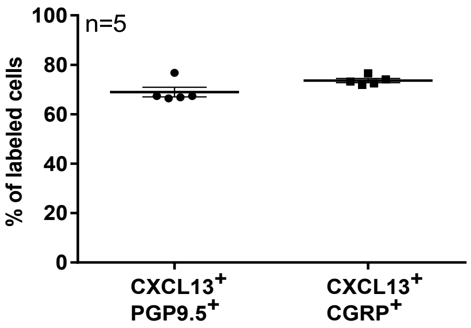

Supplementary Fig. 6 Percentages of colocalization of CXCL13 with either PGP9.5 or CGRP are comparable (69.3% and 73.3%; p=0.9358; Chi-square test; n=5 whole mounts, mean ± SEM).

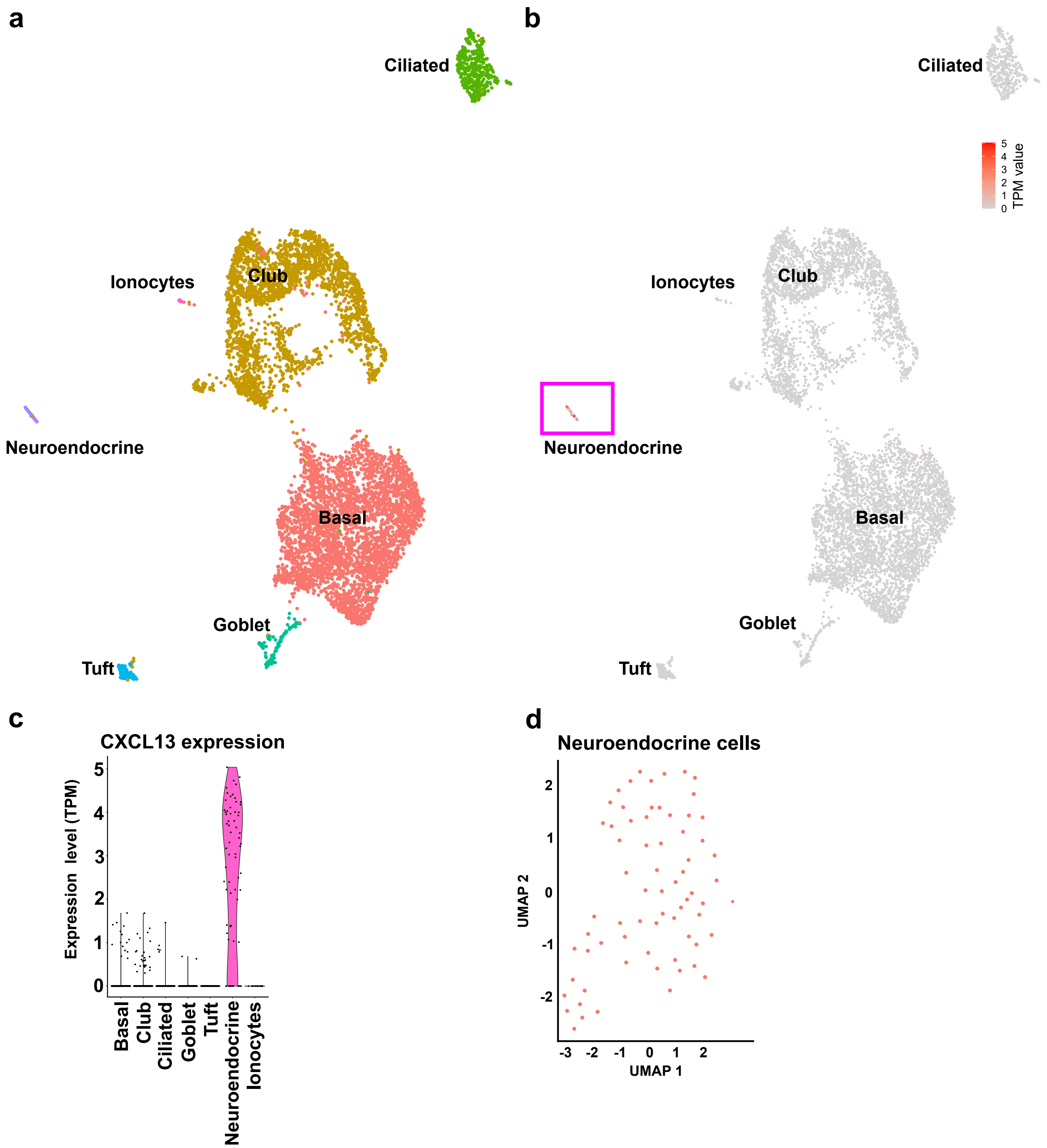

Supplementary Fig. 7 *In silico*-analysis of single cell mRNA sequencing data of tracheal neuroendocrine cells, resource data set GSE103354. (a) SPRING plot (Uniform Manifold Approximation and Projection, UMAP) shows seven distinct cell clusters, namely basal, club, ciliated, goblet, solitary cholinergic chemosensory (brush/tuft), solitary neuroendocrine cells and ionocytes. **(b and c)** SPRING and violin plots showing that *Cxcl13*-mRNA is predominantly expressed within the neuroendocrine cell cluster. **(d)** Uniform Manifold Approximation and Projection (UMAP), each dot represents a single neuroendocrine cell (n=68). There is no subclustering of neuroendocrine cells based on distinct gene expression patterns.

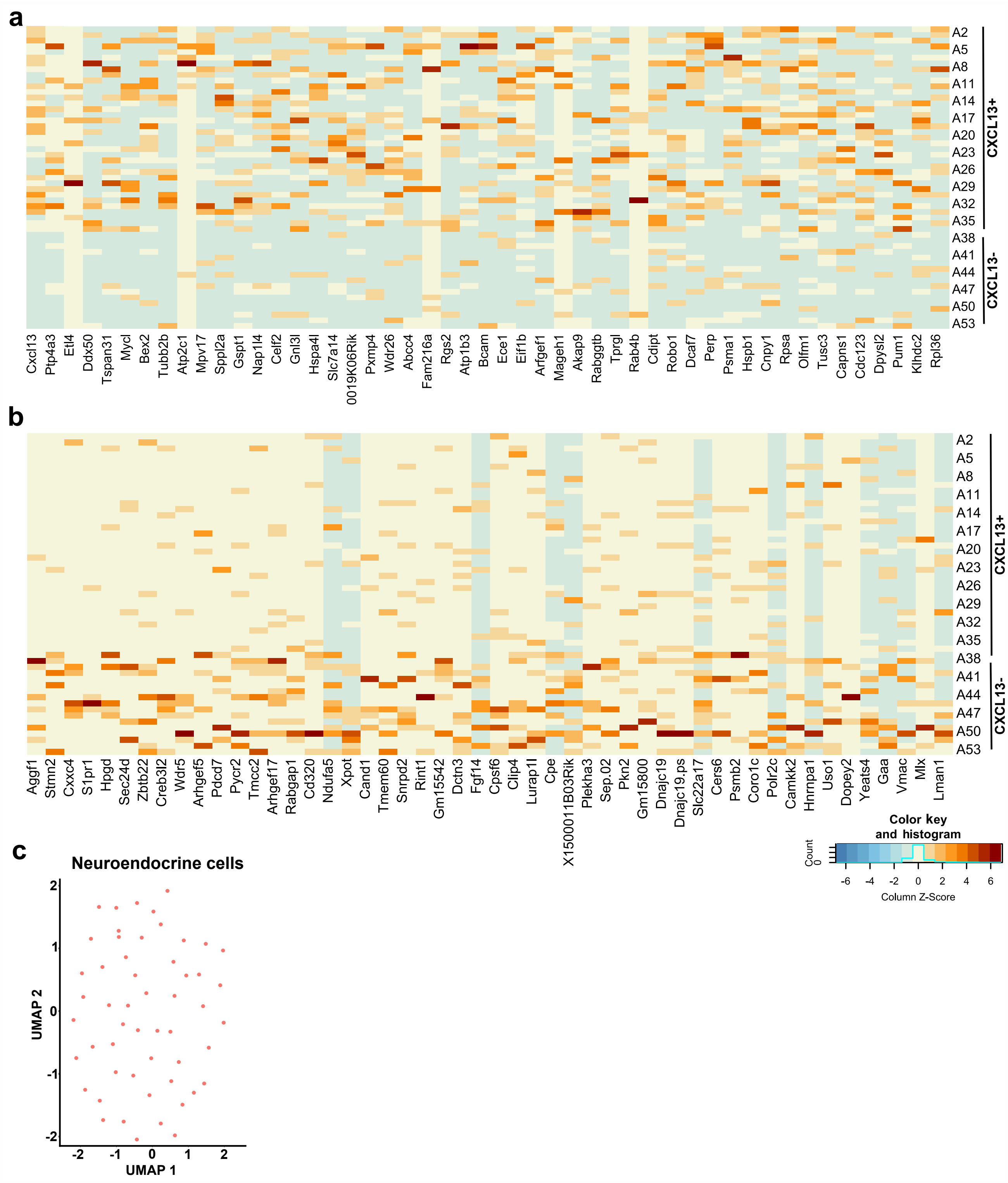

Supplementary Fig. 8 *In silico*-analysis of single cell mRNA sequencing data of tracheal neuroendocrine cells revealed no subclustering. (a and b) Heat maps showing differences in gene expression levels (fold change >2.5) between CXCL13^{+} and CXCL13^{-} neuroendocrine cells. Upper heat map **(a)**: genes upregulated in CXCL13^{+} cells. Lower heat map **(b)**: genes downregulated in CXCL13^{+} cells. **(c)** Uniform Manifold Approximation and Projection (UMAP), each dot represents a single neuroendocrine cell (n=53). There is no subclustering of neuroendocrine cells based on distinct gene expression patterns. Resource: data set GSE102580

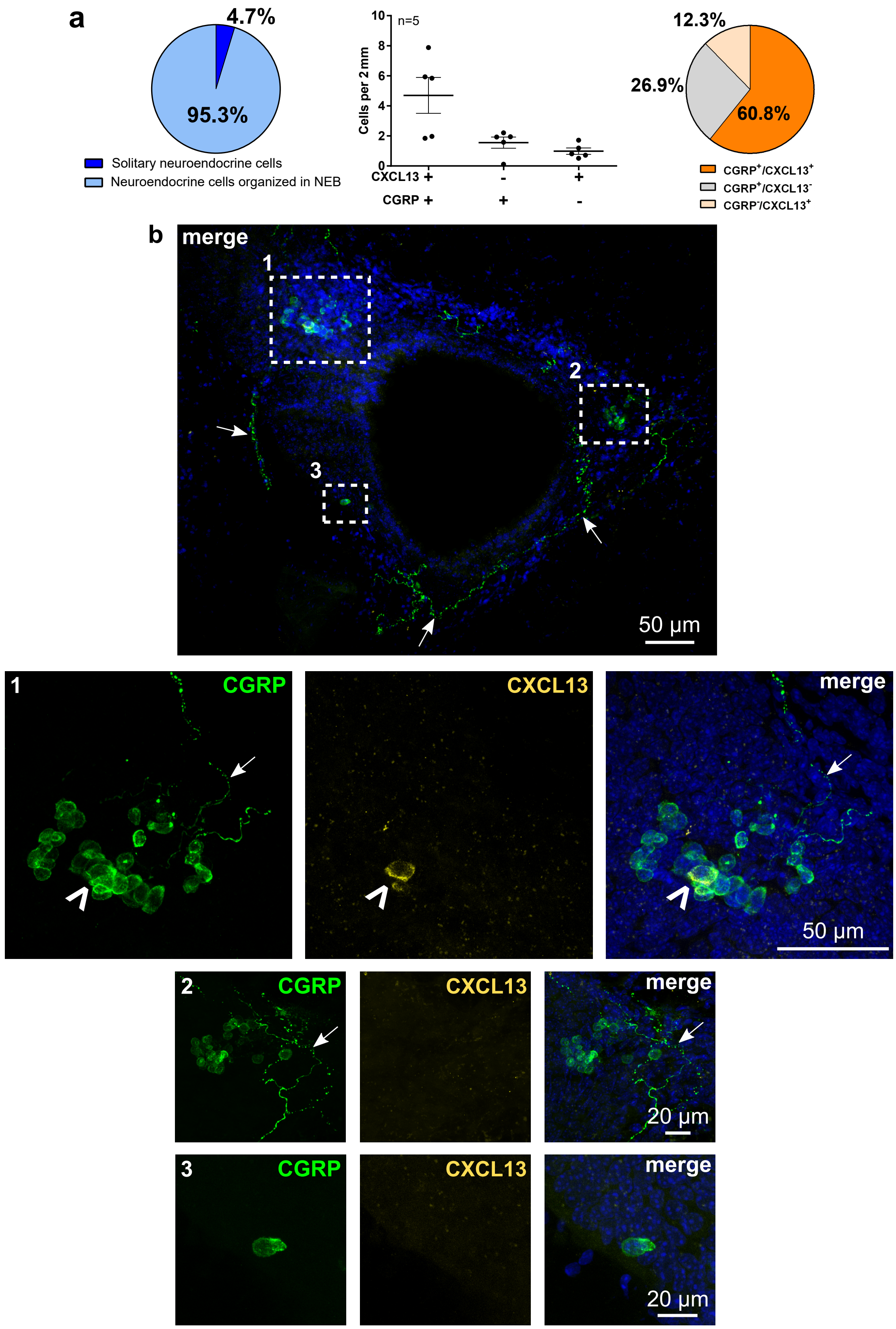

Supplementary Fig. 9 CXCL13 expression in tracheal and broncho-pulmonary neuroendocrine cells, supplementing experimental data depicted in Fig. 6. **(a)** CXCL13 expression within the bronchial epithelium. Left pie chart showing the percentages of CGRP-immunolabeled NEC in lung cryosections (total number of immunoreactive cells = 1548 from 5 animals) organized in NEB or appearing as solitary cells. Scatter plot showing numbers of immunolabeled cells per 2 mm basement membrane counted in tracheal cryosections labeled with antibodies against CGRP and CXCL13. Data points represent mean values of counts in one trachea (n=5 tracheas); mean and SEM are indicated. Percentages of immunoreactive phenotypes depicted in the right pie chart (n=413 cells). **(b)** Confocal laser scanning microscopy (Zeiss, LSM 710) of a precision-cut lung slice, double-labeled with CXCL13 and CGRP antibodies; maximum intensity projection of z-stack of confocal optical sections. Boxed areas #1 to #3 are depicted at higher magnification in the lower panels. NEB (boxed areas #1 and #2), a solitary neuroendocrine cell (boxed area #3) and nerve fibers (arrows) are stained with antibodies against CGRP. A $CGRP^{+}$ cell in NEB #1 is co-labeled with antibodies against CXCL13, whereas $CGRP^{+}$ cells of NEB #2 and the solitary neuroendocrine cell are not CXCL13-immunoreactive.

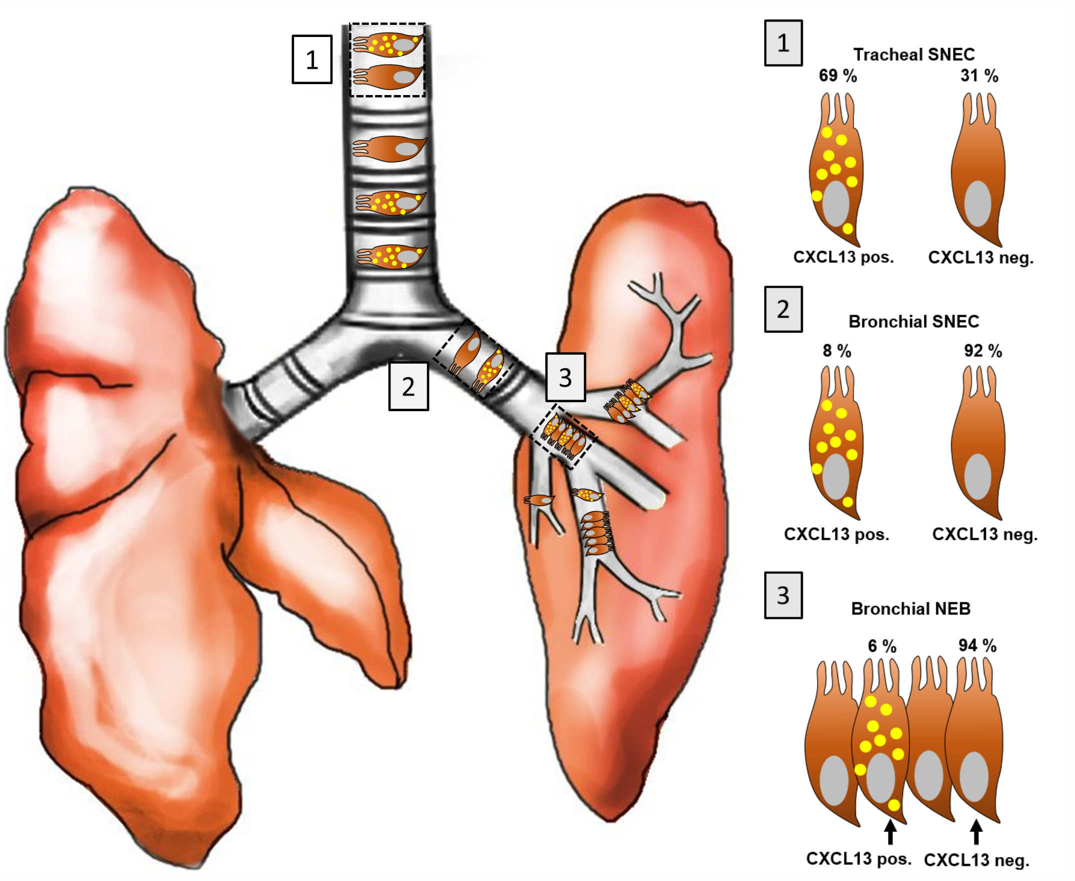

Supplementary Fig. 10 Neuroendocrine cell phenotypes based on CXCL13 expression within the tracheal and broncho-pulmonary epithelium. In the tracheal epithelium, two types of solitary neuroendocrine cells (SNEC) are present, CXCL13 pos. or CXCL13 neg. ones (1). In the bronchial epithelium, four types of neuroendocrine cells are present, CXCL13 pos. and CXCL13 neg. solitary neuroendocrine cells (2), and neuroendocrine cells (NEC) which cluster together as NEB (neuroepithelial bodies), consisting of CXCL13 neg. and CXCl13 pos. cells, or only CXCL13 neg. cells (3).

Supplementary Video 1 Tracheal whole mount staining, 3D-reconstruction of a z-stack of confocal optical sections, with two CXCL13- (magenta) and CGRP-positive (green) cells together with CGRP-imunoreactive nerve fibres. The double-positive cell in the centre comes into direct contact to a CGRP-immunoreactive nerve fibre. Nuclei labelled with DAPI (blue).
